# Local Control, Survival, and Toxicity Outcomes with High-Dose-Rate Peri-Operative Interventional Radiotherapy (Brachytherapy) in Head and Neck Cancers: A Systematic Review

**DOI:** 10.3390/jpm14080853

**Published:** 2024-08-11

**Authors:** Warren Bacorro, Bruno Fionda, Tamer Soror, Francesco Bussu, György Kovács, Luca Tagliaferri

**Affiliations:** 1 Department of Clinical Epidemiology, Faculty of Medicine and Surgery, University of Santo Tomas, 1008 Manila, Philippines; 2 Department of Radiation Oncology, Benavides Cancer Institute, University of Santo Tomas Hospital, 1008 Manila, Philippines; 3 Department of Radiation Oncology, Fondazione Policlinico Universitario Agostino Gemelli IRCCS, 00168 Rome, Italy; bruno.fionda@policlinicogemelli.it (B.F.); luca.tagliaferri@policlinicogemelli.it (L.T.); 4 Department of Radiation Oncology, University of Lübeck/UKSH-CL, 23562 Lübeck, Germany; tamer.soror@uksh.de; 5 Department of Medicine Surgery and Pharmacy, Sassari University, 07100 Sassari, Italy; fbussu@uniss.it; 6 Otolaryngology Division, Azienda Ospedaliera Universitaria di Sassari, 07100 Sassari, Italy; 7 Department of Radiological and Hematological Sciences, Università Cattolica del Sacro Cuore, 00168 Rome, Italy; gyorgy.kovacs@unicatt.it

**Keywords:** peri-operative brachytherapy, head and neck cancers, interventional radiotherapy, high dose rate

## Abstract

**Background.** Peri-operative interventional radiotherapy (POIRT) entails tumor resection, catheter implantation in the same surgery, and irradiation within the peri-operative period. It allows for maximal tumor burden reduction, better tumor bed identification, more flexible implant geometry, highly conformal irradiation, and treatment delay minimization. We reviewed the published local control, survival, toxicity, and quality of life (QOL) outcomes with POIRT for head and neck cancers (HNCs) in primary and re-irradiation settings. **Materials and Methods.** A systematic search of PubMed, Scopus, Science Direct, and other databases, supplemented by bibliography scanning and hand-searching, yielded 107 titles. Fifteen unique articles were eligible, five of which were merged with more updated studies. Of the ten remaining studies, four reported on primary POIRT, and seven reported on reirradiation POIRT. Given data heterogeneity, only qualitative synthesis was performed. **Results.** Primary POIRT in early tongue cancer results in 6-year recurrence-free (RFS) and overall survival (OS) of 92% for both; in advanced HNCs, the 9-year RFS and OS rates are 52% and 55%. Grade 1–2 toxicity is very common; grade 3–4 toxicity is rare, but grade 5 toxicity has been reported. POIRT re-irradiation for recurrent HNCs results in 5y RFS and OS rates of 37–55% and 17–50%; better outcomes are achieved with gross total resection (GTR). QOL data are lacking. **Conclusions.** Primary POIRT is safe and effective in early tongue cancers; its use in other HNC sites, especially in advanced disease, requires careful consideration. Re-irradiation POIRT is most effective and safe when combined with GTR; toxicity is significant and may be limited by careful case selection, implant planning and execution, use of smaller fraction sizes, and adherence to homogeneity constraints. **Study Registration Number.** PROSPERO Registry Number CRD42024548294.

## 1. Introduction

External beam radiotherapy (EBRT) plays a key role in the management of head and neck cancers [1]. In early disease, it is an alternative to surgery as a definitive treatment for organ preservation (oral tongue, oropharynx, larynx) or for sites that are not usually amenable to surgery (nasopharynx), and as an adjuvant treatment in resected cases with poor pathologic risk factors. In advanced, non-metastatic disease, it is given with or without concurrent chemotherapy, as an adjuvant treatment to resected cancers with poor pathologic risk factors, or for the definitive treatment of unresectable cases. Brachytherapy, or internal radiotherapy, is employed as monotherapy in lieu of or in combination with EBRT to deliver highly localized and conformal doses to allow for safe dose-escalation and maximal organ-sparing. It is employed in primarily accessible sites, whether in the definitive (lip, nasal vestibule, oral tongue, buccal, base of tongue) or adjuvant (oral tongue) setting [2].

Advances in dosimetry planning and treatment delivery, treatment planning systems, and catheter positioning techniques have contributed to reviving the role of high-dose-rate (HDR) brachytherapy and expanding its indications in head and neck cancers (HNCs), which includes post-operative IRT in primary and re-irradiation settings [3]. The term interventional radiotherapy (IRT), introduced in recent years to signify a new era in brachytherapy [3,4], will be used for the remainder of this article. A recent review of advances in head and neck HDR IRT since the publication of the GEC-ESTRO recommendations in 2017 showed increasing literature on the use of peri-operative IRT [5].

Peri-operative interventional radiotherapy (POIRT) entails catheter implantation after tumor resection during the same surgery and delivery of radiation within the peri-operative period. This requires interdisciplinary collaboration, including multidisciplinary discussion of case eligibility and management options, and pre-operative and intra-operative surgery and implant planning [4]. A systematic review on POIRT for re-irradiation in head and neck recurrences in 2017 showed promising outcomes [6]. Considering recent advances and longer follow-up of POIRT cases, the authors sought to determine outcomes with POIRT for HNCs in the primary and re-irradiation settings.

We conducted a systematic review to synthesize the evidence regarding local control, survival, toxicity, and quality of life outcomes with POIRT in HNCs in the primary and re-reirradiation settings. This will guide clinical decision-making regarding case eligibility and management planning and inform feasibility appraisal and set-up planning of POIRT programs [4].

## 2. Methods and Materials

The systematic review protocol was registered with PROSPERO (International Prospective Register of Systematic Reviews) before the systematic search and data collection (PROSPERO Registry Number CRD42024548294). The protocol development and reporting of results are per the PRISMA (Preferred Reporting Items for Systematic Reviews and Meta-Analyses) guidelines [7].

### 2.1. Eligibility

*Inclusion criteria.* Studies were included per the following criteria:

1. Study design: Clinical trials, prospective/retrospective cohorts, and case-control studies were included.
2. Population: Studies that included patients with primary or recurrent HNC, of any histology without distant metastasis, with or without prior irradiation, and treated with surgical resection and POIRT were eligible. Studies with the following co-interventions were allowed: reconstruction, external radiotherapy, and chemotherapy.
3. Outcomes: Studies that reported on any of the following outcomes were eligible: survival (recurrence-free survival, RFS; overall survival, OS), radiation toxicity (acute or late toxicity), peri-operative complications, and quality of life (QOL).
4. Setting: Studies that reported on patients treated from 1990 onwards were eligible; this restriction was intended to account for significant changes in diagnostic, medical, and surgical standards.
5. Studies with at least six months of follow-up were eligible.
6. Language: Only articles reported in the English, French, German, and Italian languages were included, given resource constraints.

*Exclusion criteria.* Studies were excluded per the following criteria:

1. Study design: Case series, case reports, and pre-clinical studies were excluded. Relevant reviews were listed for bibliography scanning. Studies that were available only as an abstract or a conference proceeding were excluded.
2. Outcomes: Studies that did not report on the above outcomes of interest, such as feasibility or dosimetric studies) were excluded.

### 2.2. Information Sources and Search Strategy

The following electronic databases were systematically searched for published studies: PubMed, Scopus, Science Direct, ASCOpubs, Cochrane Library, EBSCOhost, and Google Scholar. The International Clinical Trials Registry Platform Search Portal, ClinicalTrials.gov, and German Clinical Trials Register were searched for ongoing or recently completed trials, and the PROSPERO Registry was searched for ongoing or recently completed systematic reviews.

Search strategies were developed using medical subject headings (MeSHs) and text words related to head and neck cancers, brachytherapy, interventional radiotherapy, and peri-operative. The following is the PubMed search strategy, which was peer-reviewed and adapted to the syntax and subject headings of the other electronic databases (Supplementary File S1):

1. Head and neck cancer [MeSH Major Topic].
2. Brachytherapy [MeSH Terms].
3. Interventional radiotherapy [Title/Abstract].
4. Numbers: 2 OR 3.
5. Peri-operative [Title/Abstract].
6. Perioperative [Title/Abstract].
7. Numbers: 5 OR 6.
8. Numbers: 1 AND 4 AND 7.

The literature search was limited to human subjects.

To ensure data saturation, the electronic database search was supplemented with bibliography scanning and hand-searching for cited and citing articles. The above PubMed search strategy was updated toward the end of this review to ensure that it retrieved the most eligible studies found through any other means but indexed in PubMed.

### 2.3. Study Records

The literature search results were imported into citation manager software. Duplicates were identified and removed.

Eligibility assessments using the above criteria were performed independently by reviewers knowledgeable in the subject matter, and critical appraisal was performed using the single-reviewer approach. One reviewer (WB) screened each title or abstract; a second reviewer (BF, TS) examined the excluded titles to ensure every relevant title was included. The full text was reviewed as necessary to clarify eligibility. In the case of two or multiple reports from the same group and on a broadly similar cohort, the most recent report that best satisfied the above criteria was included.

Standardized electronic databases were used to abstract data, as itemized below. Any disagreements between the reviewers in the study selection and data abstraction processes were resolved first by discussion and, if necessary, by adjudication by a third reviewer considering two senior interventional radiation oncologists (LT, GK) and one surgeon expert in IRT (FB). Study authors were contacted to resolve any uncertainties.

### 2.4. Data Items

The following data were extracted:

1. Setting: period of treatment, country.
2. Study design and size: e.g., clinical trial, prospective cohort, retrospective cohort; number of patients.
3. Patient characteristics: median/mean age, performance status, history of irradiation.
4. Disease characteristics: histology, site, tumor size, T-stage, N-stage, setting (primary, recurrence, second primary).
5. Treatment characteristics: resection status (clear margins, microscopic residual, macroscopic residual), chemotherapy, external radiotherapy, interventional radiotherapy dose and fractionation,
6. Dosimetric parameters.
7. Outcomes: RFS, OS, incidence of acute and late toxicity, peri-operative complications, QOL.
8. Duration of follow-up: median, range.

When needed, information was derived from reported data or estimated from figures (such as Kaplan–Meier curves) in the reports. Whenever possible, data specifically on the population and intervention of interest were derived and reported. Otherwise, the data for the entire cohort were reported, along with the percentage of that cohort represented by the population and intervention of interest.

### 2.5. Outcomes and Prioritization

The primary outcome was RFS. The secondary outcomes were (1) overall survival, (2) incidence of grade ≥2 acute and late toxicity, (3) peri-operative complications, and (4) quality of life. The recurrence-free survival rate at a given interval from the initiation of treatment (e.g., 3 or 5 years) is the proportion of the cohort that lives without disease recurrence. The overall survival rate at a given interval from initiation of treatment (e.g., 3 or 5 years) is the proportion of the live cohort. Acute radiation toxicity is radiation toxicity that develops during and up to 3 months after treatment completion; late radiation toxicity is radiation toxicity that develops or persists beyond three months after treatment completion. A peri-operative complication is an adverse event in the peri-operative period (during surgery and implantation, up to one month after). Quality of life is the patient-reported health-related quality of life scores measured using standardized instruments at any given interval from treatment completion.

### 2.6. Risk of Bias Assessment

A primary reviewer (WB) assessed the risk of bias using the Critical Appraisal Skills Program (CASP) Standard Checklist for Cohort Study [8]. For each item in the tool, the procedures undertaken for each study were described, and the risk of bias per item was rated as high risk, low risk, or unclear risk. A second reviewer (BF, TS) examined the assessments, and in cases of disagreement, a third reviewer was consulted for adjudication (LT, GK, FB).

### 2.7. Data Synthesis

Baseline patient and disease characteristics, intervention, and treatment outcomes from the included studies were summarized. For toxicity rates, we used the following definition for qualifiers: very common, >20%; common, >10%; uncommon, >5%; and rare, ≤5%. The data were not appropriate for quantitative synthesis; therefore, only a qualitative synthesis was performed.

## 3. Results

### 3.1. Search Results

**Figure 1:**
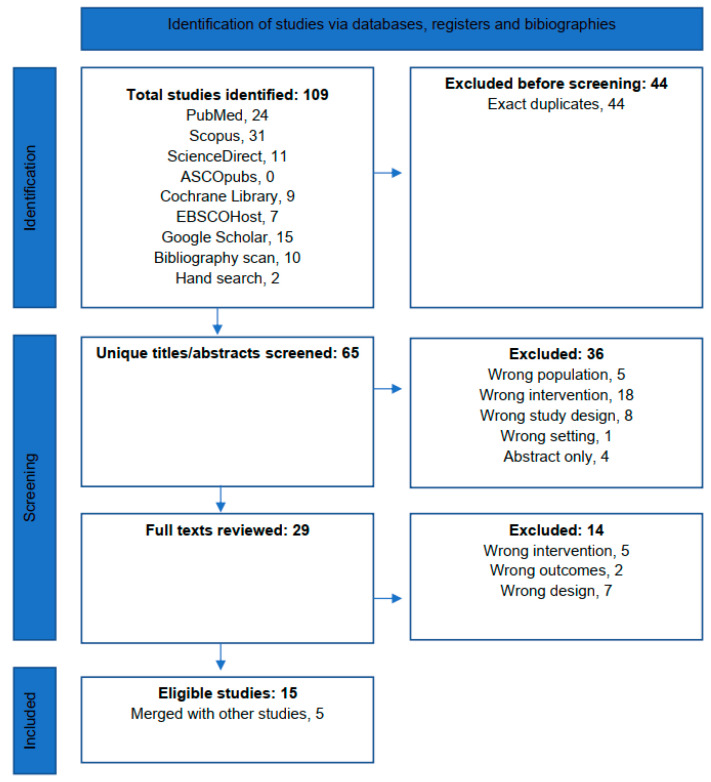
PRISMA flow diagram for the systematic search and study selection.

The systematic search and screening processes are summarized in Figure 1. The systematic search yielded 97 titles, of which, 44 duplicates were removed. A further ten titles were identified from bibliography scanning and two from hand-searching. 

### 3.2. Screening

Therefore, 65 unique titles or abstracts were screened, of which 36 were excluded because of wrong population, 5; intervention, 18; study design, 8; and setting, 1 and because of abstract-only publication, 4. A total of 29 full texts were screened, of which 14 were excluded because of wrong population, 5; outcome, 2; and design, 7. No ongoing or recently completed trials were found on the International Clinical Trials Registry Platform Search Portal or ClinicalTrials.gov or ongoing or recently completed systematic reviews on the subject in the PROSPERO Registry.

Fifteen publications were thus included in the synthesis, of which, five were merged with more updated studies [6,9,10,11,12]. Four studies reported on POIRT in the primary setting including two non-controlled trials and two retrospective cohorts [13,14]. Seven studies reported on POIRT in the re-irradiation setting including one non-controlled trial [15] and six retrospective cohorts [14,16,17,18,19,20].

## 4. Critical Appraisal

The risk of bias assessment for the included studies is summarized in Table 1 and detailed in Supplementary File S2.

*POIRT in the primary setting*. Two phase 1/2 clinical trials (Ianovski 2020, Gazatañaga 2012) [21,22] and one retrospective cohort (Potharaju 2018) [13] are associated with a low risk of bias, except for the small sample sizes, which allow for low-precision estimates. One retrospective cohort (Teudt 2014) [14] is associated with a high risk of bias given the small sample size, heterogeneity in the histologies and sequences and components of the interventions, and co-interventions allowed, which may significantly influence both the survival and toxicity outcome estimates.

*POIRT in the re-irradiation setting*. The only phase 1/2 clinical trial (Martinez-Fernandez 2017) [15] is associated with a high risk of bias due to a small sample size and a high proportion of the cohort that was treated off-protocol (de-escalated or uncompleted POIRT) because of toxicity. All six retrospective studies (Bussu 2024, Soror 2023, Ritter 2016, Teudt 2014, Rudzianskas 2012, Pellizzon 2006) [14,16,17,18,19,20] are associated with a high risk of bias mostly due to small sample size and heterogeneity in terms of the intervention, which is understandable in the re-irradiation setting, where the extent of surgery and POIRT dose must be individualized according to the site and biology of the disease and prior irradiation. Further, four of the seven studies did not provide information on the time interval to recurrence or to re-irradiation (Martinez-Fernandez 2017, Bussu 2024, Soror 2023, Teudt 2014) [14,15,16,17,18], which may significantly influence survival and toxicity outcomes. 

## 5. Scope of Extracted Data

The study information, population, and intervention characteristics are summarized in Table 2; a detailed tabulation is provided in Supplementary File S3. The survival and toxicity outcomes are summarized in Table 3 and Table 4, respectively.

The studies on POIRT in the primary setting were conducted in Europe (Germany, 1; Spain, 1), North America (Canada, 1), and Asia–Pacific (India, 1) and included cases treated from 2000 to 2017. Two reported long-term (5- and 9-year) survival outcomes and two reported short-term (3-year) survival outcomes. All reported grade ≥3 acute and late toxicity and two also reported grade 1–2 toxicity. None distinguished peri-operative complications from acute toxicity. None reported QOL.

The studies on POIRT in the re-irradiation setting were conducted in Europe (Germany, 3; Italy, 1; Lithuania, 1; Spain, 1) and South America (Brazil, 1) and included cases treated from 1994 to 2023. Three studies reported long-term (5-year) survival outcomes and four reported short-term (2- and 3-year) survival outcomes. All reported grade ≥3 acute and late toxicity and four also reported grade 1–2 toxicity. Four distinguished peri-operative complications from acute toxicity. None reported QOL.

## 6. POIRT in the Primary Setting

The included cohorts were predominantly male (male–female ratios from 0.90 to 2.89) and in the sixth to seventh decades (median ages from 52 to 62). Two studies reported oral tongue squamous cell carcinomas (OTSCCs), and one reported head and neck squamous cell carcinomas (HNSCCs), of which 35% were OTSCCs. One reported sinonasal cancer (SNC) of any histology, of which 63% were squamous cell carcinomas (SCCs).

Three studies required a gross total resection (GTR) and employed POIRT doses from 32 to 40 Gy given as 3.4 Gy to 4.0 Gy fractions twice daily with a 6 h interval within a week post-operatively. Of these, two included advanced diseases and combined POIRT with external beam radiotherapy (EBRT) doses from 45 Gy to 50 Gy; one included only early node-negative disease and did not give EBRT. On the other hand, Teudt 2014 included those who had subtotal (3%) or uncertain extent of resection (11%) and employed lower POIRT doses, from 10 Gy to 35 Gy, given as 2.5 Gy fractions twice daily 6 h apart for up to 14 days post-operatively. This was combined with higher EBRT doses, from 40 Gy to 63 Gy.

For early node-negative OTSCC treated with POIRT, excellent 6-year recurrence-free (RFS) and overall survival (OS) rates—both 92%—were reported [13]. In addition, bigger or node-positive OTSCC and good 5-year RFS and OS (69% and 59%, respectively) were reported [21]. For advanced HNCs, 3-year RFS and OS rates of 74–83% and 72–76%, respectively, and 9-year RFS and OS rates of 52% and 55% respectively, were reported [22].

Grade 1–2 acute toxicity was very common, including local edema (9–100%) and mucositis (11%). Grade 3–4 acute toxicity was rare, including fistula (5%), wound complication (2–3%), bleeding (2%), and graft failure (2%). Gaztañaga 2012 reported grade 5 bleeding in 2% of a cohort of patients treated from 2000 to 2008.

Grade 1–2 late toxicity was very common, including mucositis (17%) and wound complication, (14%). Grade 3–4 late toxicity was rare, including fibrosis (5%), soft-tissue necrosis (STN) (5%), bleeding (2%), fistula (2%), nerve damage (2%), and wound complication (2%). No osteoradionecrosis was reported. Grade 5 bleeding (4%) was reported by Gaztañaga 2012 [22].

## 7. POIRT in the Re-Irradiation Setting

The cohorts were predominantly male (male–female ratios from 2.33 to 3.29) and in the sixth to seventh decades (median ages from 53.5 to 65.6). Five studies reported predominantly oral cavity, oropharynx, or cervical node cases (~70–85%) and SCCs (80–100%). One included nasopharyngeal, ethmoid, and other sites, of which 79% were SCCs, and one reported entirely sinonasal cancers, of which 63% were SCCs. All reported predominantly recurrent cases (76–100%) with prior EBRT to median doses of 52–66 Gy. Where indicated, patients included had a recurrence or salvage therapy at median intervals of 12–32 months from the last treatment [18,19,20].

In three studies, a GTR of the recurrence was a prerequisite for POIRT. Of these, two employed POIRT without EBRT, with median POIRT doses of 40 Gy given as 4.0 Gy fractions (Martinez-Fernandez 2017) [15], or 30 Gy given as 2.5 Gy fractions, twice daily with a 6 h interval within 5–10 days post-operatively [16]. One combined a median 30 Gy EBRT dose with a median POIRT dose of 24 Gy [20]. The other four studies included cases with or without GTR (GTR, 55–85%) [14,17,18,19]. Three combined median EBRT doses of 28–50 Gy with median POIRT doses of 20–30 Gy [17,18]; one employed POIRT only to a median dose of 30 Gy [19].

For recurrent HNCs treated with POIRT re-irradiation, 3-year RFS and OS rates of 34–88% and 39–72%, respectively, and 5 y RFS and OS rates of 37–55% and 17–50%, respectively, were reported.

Grade 1–2 acute toxicity was very common, including pain (6–25%) and mucositis (11–22%). Grade 3–4 acute toxicity was common, including dysphagia (20%), pain (17%), and wound dehiscence (14%). Martinez-Fernandez et al. reported grade 5 bleeding (5%) in a cohort of patients treated from 2001 to 2015.

Grade 1–2 late toxicity was very common, including xerostomia (32%), pain (18%), dysphagia (17%), and mucositis, (17%). Grade 3–4 late toxicity was common, including fistula (19%), local ulcer (14%), and xerostomia (13%). ORN was reported in up to 5% and STN in up to 3%. Grade 5 fistula (2%) and STN (2%) were reported by Martinez-Fernandez 2017 [15].

In the re-irradiation setting, the following complications and rates were reported: wound dehiscence, 14%; flap or graft failure, 3–6%; bleeding, 3%; local infection, 3%; respiratory infection, 3%; and brachytherapy catheter dislodgement requiring replacement, 2%.

## 8. Discussion

POIRT, by combining tumor resection or debulking and IRT, allows for the maximal reduction in tumor burden, adequate coverage of target volumes at the highest risk for recurrence, and reduced doses to surrounding normal tissues. Intraoperative implantation offers an opportunity for better tumor bed identification and more flexible implant geometry; perioperative irradiation offers radiobiological advantages by limiting treatment delay. The perioperative approach could extend the application of IRT to head and neck sites that are not usually amenable to interstitial or intracavitary approaches, because of anatomical considerations, or the re-irradiation setting, where the role of newer EBRT techniques, such as stereotactic body radiotherapy, remain limited to palliative treatment [1].

### 8.1. POIRT in the Primary Setting

Potharaju et al. reviewed 73 T1-2 N0 OTSCC cases treated with either POIRT to 40 Gy over 10 fractions (n = 26) or definitive IRT to 50 Gy over 10 fractions (n = 47) [13]. In the POIRT group, ipsilateral modified radical neck dissection was performed along with tumor resection. No EBRT was given in either group. POIRT was associated with significantly better 6-year OS (92% versus 75%, *p* = 0.032), disease-free survival (DFS) (92% versus 55% vs. 92.3%, *p* = 0.002), and nodal RFS (96% vs. 68%, *p* = 0.007) when compared with definitive IRT. All nodal recurrences developed within 48 months. The volume treated to 150% of the prescribed dose (V150) was a possible risk factor for STN or ORN. Five (11%) developed ORN in the definitive IRT group, of which, one had to undergo surgery; the lower total doses and fraction sizes used for POIRT were associated with no STN or ORN. For context, meta-analyses estimate ORN incidence rates of 36% with intensity-modulated EBRT [23] and grade ≥3 ORN rates of 1% with proton therapy [24]. These suggest that POIRT could be a more effective and safer alternative to definitive IRT for early OTSCCs.

Ianovski et al. enrolled 73 T1-3 N0-3 OTSCCs (pT3, 4%; pN1, 15%; pN2, 20%; pN3, 0), of which, 41 were treated with POIRT to 34 Gy for close margins, or to 40.8 Gy for positive margins, given as 3.4 Gy fractions [21]. Unilateral or bilateral neck dissection was performed depending on clinical neck involvement. Neck EBRT was given for intermediate- or high-risk diseases, such as >2 positive nodes, extranodal extension (ENE), and bilateral neck involvement; concurrent chemotherapy was given for ENE. Despite the inclusion of pT3 and pN1-2 disease, a low local recurrence rate of 11% and good 5-year DFS and OS rates of 69% and 59%, respectively, were achieved, with rare grade 3–4 toxicity. Whether this results in improved QOL needs to be studied.

Teudt et al. reviewed primary and recurrent SNCs (node-positive disease, 63%) treated with POIRT to a median dose of 20 Gy (10–35 Gy) given as 2.5 Gy fractions, alone or combined with EBRT to a median dose of 50.4 Gy (40–63 Gy) with or without concurrent chemotherapy [14]. The cohort included 22 primary SNCs, for which an impressive 3-year DFS rate of 83% was achieved, with rare grade 3–4 toxicity.

Gaztañaga et al. treated primary and recurrent HNSCCs with POIRT to 32 Gy for negative margins, or to 40 Gy for positive margins, given as 4 Gy fractions, combined with EBRT 45 Gy [22]. The cohort included 47 primary HNSCCs (oral cavity, 52%; oropharynx, 21%), of which, 63% had pN+ disease and had concurrent chemotherapy. Failure patterns (local, 3.5%; regional, 12%; locoregional, 9%; isolated distant, 16%) and survival rates (5-year RFS and OS of 52% and 55%, both maintained at 9 years) were comparable to reference cohorts (RTOG 9501, EORTC 22931) [25,26]. However, an improved toxicity profile, as hypothesized, was not achieved, as grade ≥3 toxicity rates were comparable to the above reference cohorts. The investigators recommended delaying starting POIRT fractions 5 days post-operatively, raising dose homogeneity index (DHI) requirements (≥0.60 in the study), or prescribing absolute V150 constraints.

### 8.2. POIRT in the Re-Irradiation Setting

Five studies reported cohorts that included predominantly oral cavity, oropharynx, and neck recurrences, one reported SNC recurrences, and one included endocavitary implants for nasopharynx, nasal cavity, and ethmoid recurrences. Three required GTR, and four allowed for maximal safe resection (MSR) or subtotal resection.

With GTR and POIRT, promising 5-year RFS (43–55%) and OS (36–50%) were reported, but at the cost of significant toxicity [15,16,20]. Martinez-Fernandez et al. treated 63 HNC recurrences, (76%) or second primaries (24%) with POIRT to ≤32 Gy for negative margins or to 40 Gy for positive margins, given as 4 Gy fractions [15]. Five-year RFS (55%) and OS (36%) were promising but had high cumulative incidence rates of 16%, 27%, and 8% for grade 3, 4, and 5 toxicities, respectively. These could not be attributed to dose–volume parameters. Bussu et al. reviewed 29 HNC recurrences treated with lower POIRT doses to 30 Gy given as 2.5 Gy fractions [16]. There were no grade ≥3 toxicities; however, two-year local RFS (29%) and OS (46%) were comparatively low. Pellizzon et al. reviewed 21 HNSCCs with controlled primaries and neck recurrences; 15 had prior irradiation and were treated with POIRT to a median dose of 24 Gy combined with EBRT to a median dose of 30 Gy (25–50 Gy) [20]. Five-year RFS (43%) and OS (50%) were promising. Grade 3–4 acute (wound dehiscence, 14%) and late (local ulcer, 14%) toxicities were common but without grade 5 events.

With MSR and POIRT, inferior survival outcomes were achieved, with reported 2- or 3-year RFS and OS rates of 29–88% and 39–62%, respectively [14,18,19], and 5-year RFS and OS rates of 37% and 17%, respectively [17]. In the medium term, grade 3–4 acute and late toxicities were rare (5%) [14,18,19], but on longer follow-up, these proved to be common (>10%) [17]. Rudzianska et al. reviewed 30 HNSCC recurrences treated with either IRT alone or surgical resection and POIRT. Thirteen were treated with POIRT to 30 Gy given as 2.5 Gy fractions [19]. Better 2-year local control (77% versus 47%, *p* = 0.013) and OS (62% versus 35%, *p* = 0.035) rates were achieved with POIRT when compared with IRT alone, with rare grade 3–4 toxicity. Ritter et al. reviewed 94 HNC recurrences; 63 had prior irradiation and were retreated with POIRT to a median dose of 26 Gy (10–35 Gy) given as 2.5 Gy (2.5–4.5 Gy) fractions, [18]. In 26%, EBRT was given at a median dose of 49 Gy (30–60 Gy). The addition of systemic treatment with cetuximab–paclitaxel protocol enhanced survival rates without significantly increasing grade ≥3 acute or late toxicity when compared to POIRT ± EBRT alone in a matched-pair analysis of the two treatment subgroups. Teudt et al. reviewed 35 SNCs; 16 were recurrences and were treated with POIRT to a median dose of 20 Gy (10–35 Gy) given as 2.5 Gy fractions [14]. In 55% of the entire cohort, pre- or post-operative EBRT was given to a median of 50.4 Gy (40–63 Gy). Grade ≥3 toxicities were rare; however, 3-year RFS was low (34%). Soror et al. reviewed 60 HNC recurrences; 42 had prior irradiation and were retreated with POIRT to a median dose of 30 Gy (12–40 Gy) given as 3 Gy fractions [17]. In 12% of the entire cohort, EBRT was given to 30–50 Gy. Three-year local RFS was high (88%), but 5-year local RFS (37%) and OS (17%) rates and long-term grade 3–4 toxicity were common.

It cannot be overemphasized that combined salvage surgery and POIRT in the re-irradiation setting could be associated with significant toxicity. The most reliable estimate of toxicity rates comes from the only prospective study by Martinez-Fernandez et al., with a median follow-up of 82 months. Up to 50% of the patients experienced grade ≥3, including three grade 5, events [15]. However, their study included patients treated from 2001 to 2015. Longer-term follow-ups of more recent cohorts may better reflect the outcomes with more recent technological and clinical advancements.

### 8.3. Enhancing the Therapeutic Ratio with POIRT

In the primary setting, the use of POIRT could safely limit irradiated volumes and doses to the oral cavity and mandible among tongue cancer patients, thereby improving toxicity. It could also compensate for dosimetric limitations such as those due to bone–air interfaces in sinonasal cancers, thereby improving dosimetry and efficacy. Its use in other head and neck sites needs careful consideration, especially in more advanced diseases that require more extensive surgery and often the addition of concurrent chemotherapy to EBRT. Depending on margin status, total doses of 32 to 40 Gy, given as 3.4 to 4.0 Gy fractions twice daily, 6 h apart, are commonly used.

In the re-irradiation setting, superior survival outcomes were achieved with GTR and POIRT; therefore, GTR should be planned whenever possible. Given significant toxicity risks, cases for which GTR is unlikely on pre-operative evaluation should probably not be considered for a curative POIRT approach. Depending on margin status, total doses of 32 to 40 Gy given as 4 Gy fractions twice daily, 6 h apart, were associated with better survival outcomes; these could be given over smaller fractions (3.0–3.5 Gy) to limit toxicity.

Proper measures should be undertaken during implantation and dosimetry to limit toxicity. Mandibular clearance should be strictly observed [15,21], and multiple-plane implants should be judiciously employed to allow for better dosimetric optimization while limiting tissue trauma [15]. In early OTSCCs, the tumor bed should be implanted [13,21]. In more advanced diseases and in SNCs as well as other sites, all surgical beds considered at high risk for recurrence and a 15–20 mm safety margin must also be implanted [15,16,17,20,22]. Catheters should be uniformly implanted at 8–12 mm distances [17] unless variable catheter spacing (5–15 mm) is intended to minimize doses to organs-at-risk (closer spacing) or to deliver integrated boost (wider spacing) [14,18]. When using single-plane implants, the CTV must be kept within 5 mm of the catheters [13,21]; when a boost is intended, the CTV may be extended up to 10 mm around the catheters [14].

Higher DHI (≥0.66) [19,22], lower V150 (<13 cc) [13,15,22], and mandibular and vascular D10 cc <4 Gy per fraction [15] may need to be achieved, especially in the re-irradiation setting. Recently available inverse-planning software tools can be beneficial. If the above constraints limit POIRT doses or coverage, then a combination with highly conformal EBRT, ideally with dose summation software, should be considered. Variable catheter spacing and intensity-modulation of POIRT to allow for intended hotspots or integrated boost may be a useful approach in subtotal resection; boost doses of up to 200% of the prescribed dose should be confined entirely within the gross tumor volume [14,18]. However, intensity-modulation requires careful implant planning by an experienced team and should probably be limited to cases without prior irradiation or with low prior irradiation doses (<50 Gy) delivered by conformal or intensity-modulated techniques, with a minimum one-year interval from previous irradiation [18,19,20].

### 8.4. Study Limitations and Recommendations

The current literature on POIRT is limited to three non-controlled clinical trials and seven retrospective studies. Overall, these are associated with a high risk of bias due to small sample sizes and heterogeneity in population and intervention characteristics. This is understandable given that POIRT is an emerging intervention, re-irradiation cases are highly varied, and treatment needs to be individualized. Only survival and toxicity data were reported; QOL data are lacking.

Given the data heterogeneity, only a qualitative synthesis could be performed. Nevertheless, whenever possible, outcomes data were derived for the population and intervention of interest, and population and intervention characteristics were presented systematically to provide the reader with a good summary of the average patient and intervention for which outcomes were reported and to allow for a meaningful comparison.

To reduce variability in patient demographics, tumor profiles, and treatment methodologies, there is an urgent need for large-scale, multicenter studies adhering to standardized protocols. These studies should focus on harmonizing the collection and reporting of treatment outcomes and treatment-related toxicities. Establishing consistent criteria and methodologies across different research centers will facilitate more reliable comparisons, enhance the validity of findings, and provide clearer insights into the effectiveness and safety of treatment modalities. Our findings could help clinicians in the management of patients and in harmonizing data reporting towards collaborative research [4,27].

## 9. Conclusions

In the primary setting, POIRT is safe and effective in tongue and sinonasal cancers; its use in other head and neck sites, especially in more advanced diseases that require extensive surgery and combination with EBRT and chemotherapy, requires careful consideration and multidisciplinary planning. In the re-irradiation setting, POIRT is most effective and safe in cases for which GTR can be achieved; toxicity is significant and may be limited by careful case selection, implant planning and execution, use of smaller fraction sizes, and adherence to homogeneity constraints.

## Figures and Tables

**Table 1:** Risk of bias assessment.

Risk of Bias Assessment (CASP Checklist for Cohort Studies)												
Study ID	Research Question	Selection Bias	Measurement Bias (Exposure)	Measurement Bias (Outcomes)	Confounding Factors	Follow-Up	Magnitude of Effect	Precision of Estimate	Credibility	Empiric Congruence	Applicability	Implications to Practice
**Peri-operative interventional radiotherapy in the primary setting**												
**Non-controlled clinical trial**												
**Ianovski 2020 [21]**	Low risk	Low risk	Low risk	Low risk	Low risk	Low risk	Low risk	High risk	Low risk	Low risk	Low risk	Low risk
**Gaztañaga 2012 [22]**	Low risk	Low risk	Low risk	Low risk	Low risk	Low risk	Low risk	High risk	Low risk	High risk	Low risk	Low risk
**Retrospective cohort**												
**Potharaju 2018 [13]**	Low risk	Low risk	Low risk	Low risk	Low risk	Low risk	Low risk	High risk	Low risk	Low risk	Low risk	Low risk
**Teudt 2014 [14]**	Low risk	Low risk	High risk	Low risk	High risk	Low risk	Uncertain risk	High risk	Low risk	Low risk	High risk	Low risk
**Peri-operative interventional radiotherapy in the re-irradiation setting**												
**Non-controlled clinical trial**												
**Martínez-Fernández 2017 [15]**	Low risk	Low risk	High risk	Low risk	High risk	Low risk	Low risk	High risk	Low risk	Low risk	High risk	Low risk
**Retrospective cohort**												
**Bussu 2024 [16]**	Low risk	Low risk	Low risk	Low risk	High risk	Low risk	High risk	High risk	Low risk	Low risk	High risk	Low risk
**Soror 2023 [17]**	Low risk	High risk	High risk	Low risk	High risk	High risk	High risk	High risk	High risk	Low risk	High risk	Low risk
**Ritter 2016 [18]**	Low risk	High risk	High risk	Low risk	Low risk	High risk	Low risk	Low risk	Uncertain risk	Uncertain risk	High risk	Low risk
**Teudt 2014 [14]**	Low risk	Uncertain risk	High risk	Low risk	High risk	Low risk	Uncertain risk	High risk	Uncertain risk	Uncertain risk	High risk	Low risk
**Rudzianskas, 2012 [19]**	Low risk	Low risk	Low risk	Low risk	Low risk	Low risk	Low risk	High risk	Low risk	Uncertain risk	Low risk	Low risk
**Pellizzon, 2006 [20]**	Low risk	High risk	High risk	High risk	Low risk	High risk	Low risk	High risk	Low risk	Low risk	Low risk	Low risk

**Table 2:** Study information and population, and intervention groups and characteristics.

Study ID (References)	Country	Study Period	n (%) ^a^	M/F	Mdn Age (Range)	Site, %	Histology, %	Stage, %	Resection/ Margin Status, % /Reconstruction, %	EBRT, % /Dosing	BRT Dosing	Implant Technique and CTV		Dosimetry CTV	Start of BRT (Day Post-op)	Chemo, %/Regimen, %	
**Non-controlled clinical trials**																	
**Ianovski, 2020** [21]	Canada	Sep 2009 to Apr 2017	55 (75)	0.90 ^b^	62 ^b^ (24–92)	OT, 100 ^b^	SCC, 100 ^b^	pT1, 49 ^b^ pT2, 47 pT3, 4 pT4, 0 pN0, 65 ^b^ pN1, 15 pN2, 20	R0, 0 Close (2.1–5 mm), 58 R1, 42 Recon, 100	39 Involved neck, 55 Gy/25 F Uninvolved neck, 50 Gy/25 F	Close margins 34 Gy/10 F, 63 R1 40.8 Gy/12 F, 37	ISIRT CTV: Tumor bed		CTV: 5 mm around catheters	D3–5	34 If ENE, EBRT + concurrent weekly carboplatin 100 mg/m^2^ + taxol 40 mg/m^2^	
**Gaztañaga, 2012** [10,12,22]	Spain	Oct 2000 to Oct 2008	57 (70)	2.17	59 ^b^ (25–85)	OC, 52 ^b^ OPx, 21 HPx, 7 Neck, 18	SCC, 100	cN0, 21 cN1-2, 79 pN0, 30 pN+, 63 pNx, 7	R0 (10 mm), 12 ^b^ Close (Mdn 3.0 mm), 35 ^b^ R1, 53 ^b^	100 ^b^ 45 Gy/25 F	R0 32 Gy/8 F BID 6 h apart R1 40 Gy/10 F BID 6 h apart	ISIRT CTV: Tumor bed and all surgical bed considered recurrence risk category 2 (≥2 nodes or ENE) or 3 (R1)		CTV: Tumor bed and high-risk volumes	D2–3	63 Cisplatin–paclitaxel, 60 Cisplatin–other, 4	
**Retrospective cohort**																	
**Potharaju, 2018** [13]	India	Jan 2000 to Sep 2010	73 (36)	2.25 ^b^	52 ^b^	OT, 100 ^b^	SCC, 100 ^b^	T1, 14 ^b^ T2, 12 N0, 100 ^b^	<5 mm, x ≥5–10 mm, x Recon, 0	None	40 Gy/10 F BID 6 h apart	ISIRT, single-plane CTV: Tumor bed		CTV: 5 mm around catheters	D5–7	None	
**Teudt, 2014** [14]	Germany	Jan 2006 to Jan 2013	35 (63)	2.89 ^b^	60 ^b^	NC, 46 ^b^ PNS, 54	SCC, 63 ^b^ Adeno, 20 Other, 17	I, 17 ^b^ II, 20 III, 11 IV, 51	R0, 54 ^b^ R1, 31 R2, 3 Rx, 11 Osteosynthesis plates as needed	57 ^b^ Mdn 50.4 Gy (40–63 Gy)	Mdn 20 Gy (10–35 Gy)/2.5 Gy-F BID 6 h apart^b^	ISIRT Intensity-modulation by variable catheter spacing (5–12 mm)		CTV: Maximum 10 mm around catheters	Mdn D7 (D2–14)	31 ^b^ (chemo given only for SCC) Cisplatin, 26 Taxane, 9 Etoposide, 3	
**Adeno**, adenocarcinoma; **BID**, twice daily; **c**, clinical; **CTV**, clinical target volume; **D**, day; **EBRT**, external beam radiotherapy; **ENE**, extranodal extension; **F**, fraction; **Gy**, Gray; **h**, hour; **HPx**, hypopharynx; **IRT**, interventional radiotherapy; **ISIRT**, interstitial interventional radiotherapy; **Mdn**, median; **N**, nodal stage; **NC**, nasal cavity; **OC**, oral cavity; **OPx**, oropharynx; **OT**, oral tongue; **PNS**, paranasal sinus; **p**, pathologic; **R**, resection status; **SCC**, squamous cell carcinoma; **T**, primary tumor stage; **x**, unknown																	
a. Percentage comprising the population and intervention of interest, if from a mixed cohort. b. Separate numbers not derivable for the population or intervention of interest, numbers reported for the entire cohort.																	
**Peri-operative interventional radiotherapy in the re-irradiation setting**																	
**Study Information**			**Patient Characteristics**							**Intervention Characteristics**							
**Study ID** **(References)**	**Country**	**Study Period**	**n (%) ^a^**	**M/F**	**Mdn Age (Range)**	**Site, %**	**Histology, %**	**Setting and Stage, %**	**Prior RT, %** **/Setting, Dosing/Chemo/Time to ReRT**	**Resection/Margin Status, %** **/Reconstruction, %**		**EBRT, %** **/Dosing**	**BRT Dosing**	**Implant Technique and CTV**	**Dosimetry CTV**	**Start of BRT** **(day post-op)**	**Chemo, %** **/Regimen, %**
**Non-controlled clinical trial**																	
**Martínez-Fernández, 2017** [11,12,15,22]	Spain	Feb 2001 to Nov 2015	63 (100)	2.7	63 (26–82)	Neck, 32 OT, 24 OPx, 21 Other, 23	SCC, 95 Adeno, 2 Other, 4	*Second primary*, 24 T1-2N0, 18 T3/N+, 6 *Recurrence* 76 pN0, 38 pN+, 38 pNx, 24 ECE, 67	100 *EBRT*, 98 *IRT,* 14 *Prior surgery*, 64 *Chemo,* 32	R0 (10 mm), 11 Close (Mdn 3.0 mm), 35 R1, 54		None	≤32 Gy, 29 40 Gy, 71 *R0*: 32 Gy/8 F BID 6 h apart *R1:* 40 Gy/10 F BID 6 h apart	ISBT CTV: Tumor bed and all surgical bed considered recurrence risk category 2 (≥2 nodes or ENE) or 3 (positive margins)	CTV: Tumor bed and high-risk volumes	Mdn D4 (D0-D10)	None
**Retrospective cohort**																	
**Bussu, 2024** [6,9,16]	Italy	Dec 2010 to Jun 2023	34 (85)	2.6	Mean 64.5	*ICIRT group* NPx, 64 Ethmoid, 21 NC, 14 *ISIRT group* OC, 27 Lx, 20 HPx, 13 OPx, 13 Other, 27	*ICIRT group* SCC, 72 Adeno, 14 Other, 14 *ISIRT group* SCC, 87 Other, 13	*ICIRT group* LR, 100 (Second reRT, 3 Third reRT, 3) *ISIRT group* LR, x% RR, x%	*ICIRT group* Definitive, 64 Adjuvant, 36 *ISIRT group* Definitive, 33 Adjuvant, 67 >65 Gy, 100%	GTR, 100 *Recon* *ICBT*, 7 *ISBT*, 87		None	30 Gy/12 F BID 6 h apart	ISIRT, ICIRT CTV: Tumor bed and high-risk volumes	CTV: Tumor bed and high-risk volumes	D3–5	*ICBT*, 21 *ISBT*, 0
**Soror, 2023** [17]	Germany	Jan 2016 to Dec 2020	60 (70)	3.29 ^b^	65.6 ^b^ (15.4–92.7)	OPx, 25 ^b^ Neck, 23 OC, 23 Other, 26	SCC, 90 ^b^ Adeno, 8 Other, 2	LR, 68 ^b^ RR, 23 Second primary, 8.	70 Mdn 60 Gy (32–70) *Chemo*, 45	R0, 32 ^b^ Close margin (<5 mm), 5 R1, 18 R2, 45 *Recon* Pedicled or free flap, as indicated, x%		12 ^b^ 30–50 Gy	Mdn 30 Gy (12–40)/3 Gy-F BID 6 h apart ^b^	ISIRT CTV: Tumor bed + 15–20 mm and high-risk volumes 8–12 mm spacing	CTV: Tumor bed + 15–20 mm and high-risk volumes	D2–5	None
**Ritter, 2016** [18]	Germany	Jan 2006 to May 2013	94 (71)	Not reported	<60, 38 ^b^ ≥60, 62	OPx/NPx, 28 ^b^ OC, 26 Neck, 8 HPx/Lx, 6 Other 32	SCC, 80 ^b^ Other, 44	I-II, 33 ^b^ III-IV, 67 T1-2, 40 T3-4, 48 Tx, 10 N0, 71 N1-2, 22 N3, 3	67 Mdn 64.2 Gy (33–105) *Chemo,* 26 *Time to first recurrence* Mdn 24 mo (10–73) <3 mo, 10 ≤ 3 mo, 84	R0, 39 R1, 34 R2, 12 Rx, 6 No resection, 8 *Recon* Pedicled, microvascular or random pattern flap, as indicated, x%		26 Mdn 48.7 Gy (30–60)	Mdn 25.9 Gy (10–35)/2.5 Gy (2.5–4.5) F BID 6 h apart	ISIRT	Intensity-modulation allowed for up to 200% within macroscopic tumor OAR doses less than the reference isodose		16 ^b^ Platinum, 5 Cetuximab–taxane, 19
**Teudt, 2014** [14]	Germany	Jan 2006 to Jan 2013	35 (47)	2.89 ^b^	60 ^b^	NC, 46 ^b^ PNS, 54	SCC, 63 ^b^ Adeno, 20 Other, 17	I, 17 ^b^ II, 20 III, 11 IV, 51	Not reported	R0, 54 ^b^ R1, 31 R2, 3 Rx, 11 *Recon* Osteosynthesis plates as needed		57 ^b^ Mdn 50.4 Gy (40–63 Gy)	Mdn 20 Gy (10–35 Gy)/2.5 Gy-F BID 6 h apart	ISIRT CTV: Tumor bed Intensity-modulation by variable catheter spacing (5–12 mm)	CTV: Maximum 10 mm around catheters	Mdn D7 (D2–14)	31 (chemo given only for SCC) ^b^ Cisplatin, 26 Taxane, 9 Etoposide, 3
**Rudzianskas, 2012** [19]	Lithuania	Dec 2008 to Mar 2010	30 (43)	2.33 ^b^	59 ^b^ (41–79)	OC, 27 ^b^ NC/PNS, 13 Parotid, 3 OPx, 13 Neck, 44	SCC, 100 ^b^	LR, 57 ^b^ RR, 43	100 *Definitive*, 33 *Adjuvant*, 67 Mdn 66 Gy (50–72) *Chemo,* 30 *Time to first recurrence* Mdn 12 mo (3–19)	Not reported		None	30 Gy/12 F BID 6 h apart	ISIRT Catheter spacing 10–15 mm	3 D: CTV D90 isodose		
**Pellizzon, 2006** [20]	Brazil	Oct 1994 to Jun 2004	21 (71)	3.2 ^b^	53.5 ^b^ (31–73)	Pharynx 48 ^b^ OC, 29 Skin, 19 Neck, 5	SCC, 100 ^b^	RR, 100 ^b^	71 Mdn 52 Gy (30–66 Gy) *Chemo,* 5 *Time to salvage therapy* Mdn 32 mo (14–86)	GTR, 100 *Recon* As needed, x%		100 *ReRT subset* Mdn 30 Gy (25–50)	*ReRT subset* Mdn 24 Gy	ISIRT CTV: Tumor bed + 15–20 mm margins Single plane, 90.5% Double plane, 9.5%	CTV: Tumor bed + 5 mm	D5 (D4-D12)	3 ^b^ Platinum
**Adeno**, adenocarcinoma; **BID**, twice daily; **c**, clinical; **CTV**, clinical target volume; **D**, day; **D90**, dose received by 90% of the volume; **EBRT**, external beam radiotherapy; **ENE**, extranodal extension; **F**, fraction; **GTR**, gross total resection; **Gy**, Gray; **h**, hour; **HPx**, hypopharynx; **ICIRT**, intracavitary interventional radiotherapy; **IRT**, interventional radiotherapy; **ISIRT**, interstitial interventional radiotherapy; **LR**, local recurrence; **Lx**, larynx; **Mdn**, median; **mo**, month; **N**, nodal stage; **NC**, nasal cavity; **NPx**, nasopharynx; **OAR**, organ at risk; **OC**, oral cavity; **OPx**, oropharynx; **OT**, oral tongue; **PNS**, paranasal sinus; **p**, pathologic; **R**, resection status; **reRT**, reirradiation; **RR**, regional recurrence; **RT**, radiotherapy; **SCC**, squamous cell carcinoma; **T**, primary tumor stage; **x**, unknown; **3D**, three-dimensional																	
a. Percentage comprising the population and intervention of interest, if from a mixed cohort. b. Separate numbers not derivable for the population or intervention of interest, numbers reported for the entire cohort.																	

**Table 3:** Survival outcomes.

Peri-Operative Interventional Radiotherapy in the Primary Setting																		
	Baseline Characteristics						Intervention					Survival Outcomes						
Study ID	n, % ^a^	Mdn Age	Site %	SCC %	T1-2 %	N0 %	GTR %	EBRT %	Mdn EBRT Dose (Gy)	Mdn POIRT Dose (Gy)		Mdn FU (mo)	3y RFS %		3y OS %	5y RFS %		5y OS %
**Non-controlled clinical trials**																		
**Ianovski, 2020** [21]	55, 75	62 ^b^	OT, 100 ^b^	100 ^b^	96 ^b^	65 ^b^	100 ^b^	39	50–55 ^c^	34		25	74 ^b^		76 ^b^	69 ^b,d^		59 ^b^
**Gaztañaga, 2012** [10,12,22]	57, 70	59 ^b^	OT, 35 OPx, 21 FOM, 11 Other, 33	100	--	30	100	100	45	40		52 ^b^	--		--	52 (9y)		55 (9y)
**Retrospective cohort**																		
**Potharaju, 2018** [13]	73, 36	52 ^b^	OT, 100 ^b^	100 ^b^	100	100	100	0	0	40		74 ^b^	--		--	92 ^d^ (6y)		92 (6y)
**Teudt, 2014** [14]	35, 63	60 ^b^	PNS, 54 ^b^ NC, 46 ^b^	63 ^b^	--	37 ^b^	85 ^b^	57 ^b^	50.4	20		28 ^b^	83 ^d^		72 ^b^	--		--
*^a^ percentage of the cohort that received POIRT in the primary setting; ^b^ for the entire cohort (n); ^c^ non-overlapping with POIRT; ^d^ DFS*																		
**DFS**, disease-free survival; **EBRT**, external beam radiotherapy; **FOM**, floor of mouth; **FU**, follow up; **Gy**, Gray; **GTR**, gross total resection; **Mdn**, median; **mo**, month; **N**, nodal stage; **NC**, nasal cavity; **OPx**, oropharynx; **OS**, overall survival; **OT**, oral tongue; **PNS**, paranasal sinus; **POIRT**, peri-operative interventional radiotherapy; **RFS**, recurrence-free survival; **SCC**, squamous cell carcinoma; **T**, primary tumor stage; **y**, year																		
**Peri-operative interventional radiotherapy in the re-irradiation setting**																		
	**Baseline Characteristics**									**Intervention**				**Survival Outcomes**				
**Study ID**	**n, % ^a^**	**Mdn Age**	**Site** **%**	**SCC** **%**	**T1-2** **%**	**N0** **%**	**Rec %; Sec %**	**Mdn Prior EBRT dose (Gy)**	**Mdn Time to ReRT (mo)**	**GTR** **%**	**EBRT** **%**	**Mdn EBRT Dose (Gy)**	**Mdn POIRT Dose (Gy)**	**Mdn FU (mo)**	**3y RFS %**	**3y OS %**	**5y RFS %**	**5y OS %**
**Non-controlled clinical trials**																		
**Martínez-Fernández, 2017** [11,12,15,22]	63, 100	63	Neck, 32 OT, 24 BOT, 13 OPx, 8 Other, 23	95	--	38	76; 24	--	--	100	0	0	40	82	--	--	55	36
**Retrospective cohort**																		
**Bussu, 2024** [6,9,16]	34, 85	65	NPx, 31 ^c^ OC, 14 Ethmoid, 10 ^c^ Lx, 10 Other, 35	79	--	--	100; 0	>65	--	100	0	0	30	25	29 (2y)	46 (2y)	--	--
**Soror, 2023** [17]	60, 70	66 ^b^	OPx, 25 ^b^ OC, 23 Neck, 23 Other, 29	90 ^b^	--	--	92 ^b^; 8	60 ^b^	--	55 ^b^	12 ^b^	(30–50)	30	22	88 ^b,d^	39 ^b^	37 ^b,d^	17 ^b^
**Ritter, 2016** [18]	94, ~67	≥60	OPx/NPx, 28 ^b^ OC, 26 Neck, 9 HPx/Lx, 9 Other, 32	80 ^b^	40 ^b^	71 ^b^	100 ^b^; 0	64	24	73 ^b^	26 ^b^	49 ^b^	26 ^b^	13 ^b^	--	--	--	--
**Teudt, 2014** [14]	35, ~37	60 ^b^	PNS, 54 ^b^ NC, 46 ^b^	63 ^b^	--	37 ^b^	--	--	--	85 ^b^	55 ^e^	28 ^b^	20	28 ^b^	34 ^f^	72 ^b^	--	--
**Rudzianskas, 2012** [19]	30, 43	59 ^b^	Neck, 44 ^b^ OC, 27 OPx, 13 Other, 16	100 ^b^	--	--	100 ^b^; 0	66 ^b^	~12 ^g^	--	0	0	30 ^b^	16 ^b^	53 ^b,f^ (2y)	62 (2y)	--	--
**Pellizzon, 2006** [20]	21, 71	54 ^b^	Pharynx, 47 ^b^ OC, 29 Other, 24	100 ^b^	(rT0)	0 ^b^	100 ^b^; 0	52	~32 ^h^	100	100 ^b^	30	24	36 ^b^	--	--	43 ^b,d^	50 ^b^
*^a^ percentage of the cohort (N) that received POIRT in the re-irradiation setting; ^b^ for the entire cohort (n); ^c^ endocavitary; ^d^ local RFS; ^e^ including pre-op or post-op EBRT; ^f^ DFS; ^g^ time to recurrence; ^h^ time to salvage therapy*																		
**BOT**, base of tongue; **EBRT**, external beam radiotherapy; **FU**, follow up; **Gy**, Gray; **GTR**, gross total resection; **HPx**, hypopharynx; **Lx**, larynx; **Mdn**, median; **mo**, month; **N**, nodal stage; **NC**, nasal cavity; **NPx**, nasopharynx; **OC**, oral cavity; **OPx**, oropharynx; **OS**, overall survival; **OT**, oral tongue; **PNS**, paranasal sinus; **POIRT**, peri-operative interventional radiotherapy; **Rec**, recurrence; **ReRT**, reirradiation; **RFS**, recurrence-free survival; **SCC**, squamous cell carcinoma; **Sec**, secondary primary; **T**, primary tumor stage; **y**, year																		

**Table 4:** Toxicity outcomes.

Peri-Operative Interventional Radiotherapy in the Primary Setting																			
	Baseline Characteristics				Intervention								Toxicity Outcomes						
Study ID	n, % ^a^	Mdn Age	Site %		GTR %	Recon %	EBRT %	Mdn EBRT Dose (Gy)	Mdn POIRT Dose (Gy)	Dosimetry Constraints	POIRT Start (Day PO)		Mdn FU (mo)	Acute			Late		
														Grade 1–2 %	Grade 3–4 %	Grade 5 %	Grade 1–2 %	Grade 3–4 %	Grade 5 %
**Non-controlled clinical trials**																			
**Ianovski, 2020** [21]	55, 75	62 ^b^	OT, 100 ^b^		100 ^b^	100 ^b^	39	50–55 ^c^	34	--	3–5		25	Glossitis, 100	Bleeding, 2	0	Local pain, 7	0	0
**Gaztañaga, 2012** [10,12,22]	57, 70	59 ^b^	OT, 35 OPx, 21 FOM, 11 Other, 33		100	--	100	45	40	DHI ≥ 0.6	2–3		52 ^b^	--	Fistula, 5 ^b^ Bleeding, 2 ^b^ Graft failure, 2 ^b^ Wound complication, 2 ^b^	Bleeding, 2 ^b^	--	Fibrosis, 5 ^b^ STN, 5 ^b^ Bleeding, 2 ^b^ Fistula, 2 ^b^ Nerve damage, 2 ^b^ Wound complication, 2 ^b^ ORN, 0 ^b^	Bleeding, 4 ^b^
**Retrospective cohort**																			
**Potharaju, 2018** [13]	73, 36	52 ^b^	OT, 100 ^b^		100	0	0	0	40	--	5–7		74 ^b^	--	--	0	--	STN, 0 ORN, 0	0
**Teudt, 2014** [14]	35, 63	60 ^b^	PNS, 54 ^b^ NC, 46 ^b^		85 ^b^	--	57 ^b^	50.4	20	--	2–14		28 ^b^	Mucosal crusting, 11 ^b^ Peri-orbital edema, 9 ^b^ Allodynia, 6 ^b^ Wound complication, 6 ^b^ Alopecia, 3 ^b^ Dysesthesia, 3 ^b^ Epiphora, 3 ^b^ Fatigue, 3 ^b^ Flushing, 3 ^b^	Wound complication, 3 ^b^	0 ^b^	Mucosal crusting, 17 ^b^ Wound complication, 14 ^b^ Dysgeusia due to hyposmia, 14 ^b^ Allodynia, 6 ^b^ Epiphora, 6 ^b^ Peri-orbital Edema, 6 ^b^ Eustachian tube dysfunction, 3 ^b^	0 ^b^	0 ^b^
*^a^ percentage of the cohort that received POIRT in the primary setting; ^b^ for the entire cohort (n); ^c^ non-overlapping with POIRT*																			
**DHI**, dose homogeneity index; **EBRT**, external beam radiotherapy; **FOM**, floor of mouth; **FU**, follow up; **GTR**, gross total resection; **Gy**, Gray; **Mdn**, median; **N**, nodal stage; **NC**, nasal cavity; **OPx**, oropharynx; **ORN**, osteoradionecrosis; **OT**, oral tongue; **PNS**, paranasal sinus; **PO**, post-op; **POIRT**, peri-operative interventional radiotherapy; **STN**, soft tissue necrosis; **T**, primary tumor stage																			
**Peri-operative interventional radiotherapy in the re-irradiation setting**																			
	**Baseline Characteristics**					**Intervention**							**Toxicity Outcomes**						
**Study ID**	**n, % ^a^**	**Mdn Age**	**Site** **%**	**Mdn Prior EBRT Dose (Gy)**	**Mdn Time to ReRT (mo)**	**GTR** **%**	**Recon** **%**	**EBRT** **%**	**Mdn EBRT Dose (Gy)**	**Mdn POIRT Dose (Gy)**	**Dosimetry Constraints**	**POIRT Start (Day PO)**	**Mdn FU (mo)**	**Acute**			**Late**		
														**Grade 1–2** **%**	**Grade 3–4** **%**	**Grade 5** **%**	**Grade 1–2** **%**	**Grade 3–4** **%**	**Grade 5** **%**
**Non-controlled clinical trials**																			
**Martínez-Fernández, 2017** [11,12,15,22]	63, 100	63	Neck, 32 OT, 24 BOT, 13 OPx, 8 Other, 23	--	--	100	--	0	0	40	V150 (6 Gy) <13 cc Mandibular/vascular D10 cc <4 Gy	0–10	82	--	Wound dehiscence, 8 ^b^ Graft failure, 6 ^b^ Bleeding, 5 ^b^	Delayed bleeding, 3 ^b^ Post-op bleeding, 2 ^b^ Post-op mortality before BRT completion, 2 ^b^	--	Fistula, 19 ^b^ ORN, 5 ^b^ STN, 3 ^b^ Dysphagia, 3 ^b^ Fibrosis, 3 ^b^ Nerve damage, 3 ^b^	Fistula, 2 ^b^ STN, 2 ^b^
**Retrospective cohort**																			
**Bussu, 2024** [6,9,16]	34, 85	65	NPx, 31 ^c^ OC, 14 Ethmoid, 10 ^c^ Lx, 10 Other, 35	>65	--	100	94	0	0	30	QUANTEC	3–5	25	Cranial neuropathy, 3 Graft failure, 3	0	0	0	0	0
**Soror, 2023** [17]	60, 70	66 ^b^	OPx, 25 ^b^ OC, 23 Neck, 23 Other, 29	60 ^b^	--	55 ^b^	--	12 ^b^	(30–50)	30	--	2–5	22	Pain, 25 ^b^ Mucositis, 22 ^b^ Xerostomia, 15 ^b^ Dysphagia, 13 ^b^ Hypogeusia, 8 ^b^ Hyposmia, 3 ^b^ Bleeding, 3 ^b^	Dysphagia, 20 ^b^ Pain, 17 ^b^ Xerostomia, 10 ^b^ Hyposmia, 3 ^b^ Local infection, 3 ^b^ Respiratory infection, 3 ^b^ Hypogeusia, 2 ^b^ Mucositis, 2 ^b^	0	Xerostomia, 32 ^b^ Pain, 18 ^b^ Dysphagia, 17 ^b^ Hypogeusia, 15 ^b^ Mucositis, 10 ^b^ Hyposmia, 3 ^b^	Xerostomia, 13 ^b^ Dysphagia, 10 ^b^ Pain, 8 ^b^ Hyposmia, 5 ^b^ Mucositis, 5 ^b^ Hypogeusia, 3 ^b^ ORN, 2 ^b^ STN, 2 ^b^	0
**Ritter, 2016** [18]	94, ~67	≥60	OPx/NPx, 28 ^b^ OC, 26 Neck, 9 HPx/Lx, 9 Other, 32	64	24	73 ^b^	--	26 ^b^	49 ^b^	26 ^b^	GTV boost up to 200% allowed OAR doses less than reference isodose	--	13 ^b^	--^e^	--^e^	0	STN, 0 ORN, 0	STN, 0 ORN, 0	0
**Teudt, 2014** [14]	35, ~37	60 ^b^	PNS, 54 ^b^ NC, 46 ^b^	--	--	50.4	--	20	28 ^b^	20	--	2–14	28 ^b^	Mucosal crusting, 11 ^b^ Peri-orbital edema, 9 ^b^ Allodynia, 6 ^b^ Wound complication, 6 ^b^ Alopecia, 3 ^b^ Dysesthesia, 3 ^b^ Epiphora, 3 ^b^ Fatigue, 3 ^b^ Flushing, 3 ^b^	Wound complication, 3 ^b^	0 ^b^	Mucosal crusting, 17 ^b^ Dysgeusia due to Hyposmia, 14 ^b^ Wound complication, 14 ^b^ Allodynia, 6 ^b^ Epiphora, 6 ^b^ Peri-orbital edema, 6 ^b^ Eustachian tube dysfunction, 3 ^b^	0 ^b^	0 ^b^
**Rudzianskas, 2012** [19]	30, 43	59 ^b^	Neck, 44 ^b^ OC, 27 OPx, 13 Other, 16	66 ^b^	~12 ^c^	--	--	0	0	30 ^b^	--	--	16 ^b^	Fibrosis, 6 ^b^	Wound complication, 3 ^b^ Bleeding, 0 ^b^	0 ^b^	Dysphagia, 3 ^b^ Hoarseness, 3 ^b^	ORN, 3 ^b^	0 ^b^
**Pellizzon, 2006** [20]	21, 71	54 ^b^	Pharynx, 47 ^b^ OC, 29 Other, 24	52	~32 ^d^	100	--	100 ^b^	30	24	Dmax ≤135% Skin dose <60%	4–12	36 ^b^	--	Wound dehiscence, 14 ^b^ Subcutaneous infection, 5 ^b^	0 ^b^	--	Local ulcer, 14 ^b^ Neck fibrosis, 5 ^b^ STN, 0 ^b^ ORN, 0 ^b^	0 ^b^
*^a^ percentage of the cohort (N) that received POIRT in the re-irradiation setting; ^b^ for the entire cohort (n); ^c^ time to recurrence; ^d^ time to salvage therapy; ^e^ reported overall grade 1–2 and grade 3 toxicity rates of 17% and 10%, chronicity not specified*																			
**BOT**, base of tongue; **cc**, cubic centimeter; **Dmax**, maximum dose; **EBRT**, external beam radiotherapy; **FOM**, floor of mouth; **FU**, follow up; **GTR**, gross total resection; **GTV**, gross tumor volume; **Gy**, Gray; **Lx**, larynx; **Mdn**, median; **N**, nodal stage; **NC**, nasal cavity; **NPx**, nasopharynx; **OC**, oral cavity; **OAR**, organ at risk; **OPx**, oropharynx; **ORN**, osteoradionecrosis; **OS**, overall survival; **OT**, oral tongue; **PNS**, paranasal sinus; **PO**, post-op; **POIRT**, peri-operative interventional radiotherapy; **QUANTEC**, Quantitative Analysis of Normal Tissue Effects in the Clinic; **ReRT**, re-irradiation; **RFS**, recurrence-free survival; **SCC**, squamous cell carcinoma; **STN**, soft tissue necrosis; **T**, primary tumor stage; **Vn**, volume receiving n% of the prescribed dose																			

## Data Availability

Data sharing does not apply to this article as no datasets were generated or analyzed during the current study.

## Supplementary Materials

The following supporting information can be downloaded at https://www.mdpi.com/article/10.3390/jpm14080853/s1. Supplementary File S1. Detailed search strategy; Supplementary File S2. Detailed risk of bias Assessment; Supplementary File S3. Detailed study population and intervention characteristics.
