# Supplementary material for: Local Control, Survival, and Toxicity Outcomes with High-Dose-Rate Peri-Operative Interventional Radiotherapy (Brachytherapy) in Head and Neck Cancers: A Systematic Review
Source: J Pers Med. 2024 Aug 11;14(8):853. doi: 10.3390/jpm14080853 (PMC11355512; doi:10.3390/jpm14080853)
Supplement: Supplementary file 1 [file jpm-14-00853-s001.zip › S1 Appendix A Search Strategy.docx]

**SUPPLEMENTARY FILE S1**

**SEARCH STRATEGY AND YIELD**

| **Source** | **Search Strategy / Search Terms** | **Date/Time of Search** | **Results** | |
| --- | --- | --- | --- | --- |
|  |  |  | **Yield** | **Eligible** |
| ***Publication database*** | | | | |
| PubMed | 1. head and neck cancer [MeSH Major Topic]  2. brachytherapy [MeSH Terms]  3. interventional radiotherapy [Title/Abstract]  4. 2 OR 3  5. peri-operative [Title/Abstract]  6. perioperative [Title/Abstract]  7. 5 OR 6  8. 1 AND 4 AND 7  Filters: Humans | May 26, 2024  12:00PM | 24 |  |
| Scopus | TITLE-ABS-KEY("head and neck cancer") AND TITLE-ABS-KEY( brachytherapy OR "interventional radiotherapy" ) AND TITLE-ABS-KEY( peri-operative OR perioperative )  Filters: None | May 26, 2024  12:00PM | 31 |  |
| ScienceDirect | "head and neck cancer" AND ( brachytherapy OR "interventional radiotherapy" ) AND ( peri-operative OR perioperative )  Filters: None | May 26, 2024  12:00PM | 11 |  |
| ASCOpubs | SUBJECT "head and neck cancers" AND TITLE ( brachytherapy OR "interventional radiotherapy" ) AND TITLE ( peri-operative OR perioperative )  Filters: None | May 26, 2024  12:00PM | 0 |  |
| Cochrane Library | TITLE-ABS-KEY("head and neck cancer") AND TITLE-ABS-KEY( brachytherapy OR "interventional radiotherapy" ) AND TITLE-ABS-KEY( peri-operative OR perioperative )  Filters: None | May 26, 2024  12:00PM | 9 |  |
| EBSCOHost | SU ( head and neck cancer ) AND SU ( brachytherapy OR "interventional radiotherapy" ) AND AB ( peri-operative OR perioperative )  Filters: Medical databases | May 26, 2024  12:00PM | 7 |  |
| Google Scholar | allintitle: (peri-operative OR perioperative) AND (brachytherapy OR "interventional radiotherapy") AND ("head and neck" OR nasopharynx OR oropharynx OR tonsil OR palate OR hypopharynx OR larynx OR "oral cavity" OR tongue OR lip OR buccal OR "paranasal sinus" OR maxilla OR ethmoid OR sphenoid OR "nasal vestibule" OR nose OR “nasal cavity" OR orbit OR ear OR mandible OR alveol* OR neck ) | May 26, 2024  12:00PM | 15 |  |
| Bibliography scan |  | May 26, 2024  12:00PM | 10 |  |
| Hand search |  | June 10, 2024  12:00PM | 2 |  |
| *Exact duplicates* | | | 44 |  |
| *Unique studies* | | | 65 | 15 |
