# Supplementary material for: Local Control, Survival, and Toxicity Outcomes with High-Dose-Rate Peri-Operative Interventional Radiotherapy (Brachytherapy) in Head and Neck Cancers: A Systematic Review
Source: J Pers Med. 2024 Aug 11;14(8):853. doi: 10.3390/jpm14080853 (PMC11355512; doi:10.3390/jpm14080853)
Supplement: Supplementary file 1 [file jpm-14-00853-s001.zip › S2 Appendix B Risk of Bias Assessment.docx]

**SUPPLEMENTARY FILE S5**

**Risk of Bias Assessment**

**Summary Tables**

| **Risk of Bias Assessment (CASP Checklist for Cohort Studies)** |
| --- |

| **Study ID** | **Research Question** | **Selection bias** | **Measurement bias (exposure)** | **Measurement bias (outcomes)** | **Confounding factors** | **Follow-up** | **Magnitude of effect** | **Precision of estimate** | **Credibility** | **Empiric congruence** | **Applicability** | **Implications to Practice** |
| --- | --- | --- | --- | --- | --- | --- | --- | --- | --- | --- | --- | --- |
| **Peri-operative interventional radiotherapy in the primary setting** | | | | | | | | | | | | |
| **Non-controlled clinical trial** | | | | | | | | | | | | |
| Ianovski 2020 | **Low risk** | **Low risk** | **Low risk** | **Low risk** | **Low risk** | **Low risk** | **Low risk** | **High risk** | **Low risk** | **Low risk** | **Low risk** | **Low risk** |
| Gaztañaga 2012 | **Low risk** | **Low risk** | **Low risk** | **Low risk** | **Low risk** | **Low risk** | **Low risk** | **High risk** | **Low risk** | **High risk** | **Low risk** | **Low risk** |
| **Retrospective cohort** | | | | | | | | | | | | |
| Potharaju 2018 | **Low risk** | **Low risk** | **Low risk** | **Low risk** | **Low risk** | **Low risk** | **Low risk** | **High risk** | **Low risk** | **Low risk** | **Low risk** | **Low risk** |
| Teudt 2014 | **Low risk** | **Low risk** | **High risk** | **Low risk** | **High risk** | **Low risk** | **Uncertain risk** | **High risk** | **Low risk** | **Low risk** | **High risk** | **Low risk** |
| **Peri-operative interventional radiotherapy in the re-irradiation setting** | | | | | | | | | | | | |
| **Non-controlled clinical trial** | | | | | | | | | | | | |
| Martínez-Fernández 2017 | **Low risk** | **Low risk** | **High risk** | **Low risk** | **High risk** | **Low risk** | **Low risk** | **High risk** | **Low risk** | **Low risk** | **High risk** | **Low risk** |
| **Retrospective cohort** | | | | | | | | | | | | |
| Bussu 2024 | **Low risk** | **Low risk** | **Low risk** | **Low risk** | **High risk** | **Low risk** | **High risk** | **High risk** | **Low risk** | **Low risk** | **High risk** | **Low risk** |
| Soror 2023 | **Low risk** | **High risk** | **High risk** | **Low risk** | **High risk** | **High risk** | **High risk** | **High risk** | **High risk** | **Low risk** | **High risk** | **Low risk** |
| Ritter 2016 | **Low risk** | **High risk** | **High risk** | **Low risk** | **Low risk** | **High risk** | **Low risk** | **Low risk** | **Uncertain risk** | **Uncertain risk** | **High risk** | **Low risk** |
| Teudt 2014 | **Low risk** | **Uncertain risk** | **High risk** | **Low risk** | **High risk** | **Low risk** | **Uncertain risk** | **High risk** | **Uncertain risk** | **Uncertain risk** | **High risk** | **Low risk** |
| Rudzianskas, 2012 | **Low risk** | **Low risk** | **Low risk** | **Low risk** | **Low risk** | **Low risk** | **Low risk** | **High risk** | **Low risk** | **Uncertain risk** | **Low risk** | **Low risk** |
| Pellizzon, 2006 | **Low risk** | **High risk** | **High risk** | **High risk** | **Low risk** | **High risk** | **Low risk** | **High risk** | **Low risk** | **Low risk** | **Low risk** | **Low risk** |
|  | | | | | | | | | | | | |

**Detailed Critical Appraisal**

| **Peri-operative interventional radiotherapy in the primary setting** |
| --- |

| **Study ID** | **Research Question** | **Selection bias** | **Measurement bias (exposure)** | **Measurement bias (outcomes)** | **Confounding factors** | **Follow-up** | **Magnitude of effect** | **Precision of estimate** | **Credibility** | **Empiric congruence** | **Applicability** | **Implications to Practice** |
| --- | --- | --- | --- | --- | --- | --- | --- | --- | --- | --- | --- | --- |
| **Non-controlled clinical trial** | | | | | | | | | | | | |
| Ianovski, 2020 | **Low risk** | **Low risk** | **Low risk** | **Low risk** | **Low risk** | **Low risk** | **Low risk** | **High risk** | **Low risk** | **Low risk** | **Low risk** | **Low risk** |
|  | The study prospectively assessed the feasibility and effectiveness, in terms of recurrence, survival, and toxicity outcomes, of POIRT with or without EBRT for resected, nonmetastatic OTSCC with positive or narrow margins. | From September 2009 to April 2017, 55 pts with biopsy proven OTSCC (T1–3, N0–2, M0) were recruited into the study. The definitions used for surgical margin adequacy are standard. Forty-one had POIRT. | The surgery, POIRT, EBRT and ChT protocols were described adequately.  All pts had a partial glossectomy with or without neck dissection, as appropriate. | Survival events were per standard definitions.   Toxicity criteria was evaluated per standard criteria (RTOG acute/late radiation morbidity scoring schema).  The Kaplan-Meier method was used in the survival analyses. | Pertinent pt and disease variables were summarized and accounted for in the analyses and discussion. | The FU schedule and modalities were described adequately. the median FU length (25 months) was adequate for the reported outcome measures. All pts were accounted for. Less than 10% (2%) was treated off-protocol. | The 3y survival and disease control rates are acceptable, given the proportion of pts with pN+ disease (35%).   The 3y OS and DSS were 75.6% and 82.3%, respectively. The 3y DFS, LC, and LRC were 74.3, 86.3, and 77.7%, respectively.  The toxicity rates are acceptable (grade 3-4 acute toxicity, 2.4%; grade 1-2 late toxicity, 7.3%). No grade 5 toxicity. | CIs were not reported.   The sample size (n=41) allows for estimates with low to moderate precision. | The 3y DSS and 3y LRC are comparable to historical cohorts of early T-stage OTSCC treated with adjuvant EBRT ± concurrent ChT. | The results are compatible with the notion that a more limited radiotherapy volume may suffice for close or positive margins, especially in the absence of other risk factors. | The surgical and BRT techniques are current.   The adjuvant EBRT used more hypofractionated regimens than are usually used. The concurrent ChT agents (carboplatin + taxol) used are also not the agents commonly used.  It is not clear which staging system was used. This may affect the staging profile of the cohort but will not likely affect the protocol-defined management (margin status for IRT nodal status status for EBRT and ChT). | The study provides prospective data on the medium-term recurrence, survival, and toxicity rates with POIRT ± EBRT for resected, nonmetastatic OTSCC with close or positive margins. |
| Gaztañaga, 2012 | **Low risk** | **Low risk** | **Low risk** | **Low risk** | **Low risk** | **Low risk** | **Low risk** | **High risk** | **Low risk** | **High risk** | **Low risk** | **Low risk** |
|  | The study prospectively assessed the disease control, survival, and complication rates with POIRT with or without EBRT for resected, nonmetastatic primary or recurrent HNSCC. | From October 2000 to October 2008, 57 pts with primary or recurrent HNSCC were recruited into the study. The definitions used for surgical margin adequacy are standard. Forty had primary disease and had POIRT + EBRT. | The surgery, POIRT, EBRT and ChT protocols were described adequately.  All pts had a GTR.  Less than 10% (2%) was treated off-protocol. | Survival events were per standard definitions.   Toxicity criteria was evaluated per standard criteria (RTOG acute/late radiation morbidity scoring schema).  Toxicities strongly related to the surgery and that occurred before completion of the POIRT were excluded from the POIRT toxicity analysis.  The Kaplan-Meier method was used in the survival analyses. | Pertinent pt and disease variables were summarized and accounted for in the analyses and discussion. | The FU schedule and modalities were described adequately. the median FU length (52 months) was adequate for the reported outcome measures. All pts were accounted for. | The 5y survival and disease control rates are acceptable, given the proportion of pts with cN+ (79%) or pN+ (63.2%) disease.   The 5y OS and DFS were 55.2% and 52.4%, respectively, comparable to historical cohorts treated with adjucant EBRT ± ChT (RTOG 9501, EORTC 22931).  The 5y cumulative incidence of severe (grade ≥3 toxicity) was 37.8%, including grade 5 acute (2.5%) and grade 5 late (3.5%) bleeding. | CIs were not reported.   The sample size (n=40) allows for estimates with low to moderate precision. | The 5y OS, DFS and toxicity rates are comparable to historical cohorts of HNSCC treated with adjuvant EBRT ± ChT (RTOG 9501, EORTC 22931). | The results are not compatible with the notion that more limited irradiation may result in better tolerance or toxicity profile. The authors attribute this to a need to refine POIRT protocols. | The surgical, BRT and EBRT techniques are current. | The study provides prospective data on the long-term disease control, survival, and complication rates with POIRT + EBRT for resected, nonmetastatic HNSCC. |
| **Retrospective cohort** | | | | | | | | | | | | |
| Potharaju 2018 | **Low risk** | **Low risk** | **Low risk** | **Low risk** | **Low risk** | **Low risk** | **Low risk** | **High risk** | **Low risk** | **Low risk** | **Low risk** | **Low risk** |
|  | The study retrospectively evaluated the long-term locoregional control, survival, and toxicity outcomes of exclusive HDR BRT and POIRT in early OTSCC. | Between January 2000 and September 2010, pts with UICC 17th edition (2009) clinically staged T1/T2 N0 M0 OTSCC were treated with exclusive HDR BRT or POIRT.   The POIRT group included 26 pts treated with a partial glossectomy with ipsilateral neck dissection, and POIRT. | The surgery and BRT protocols were described adequately.  All pts in the POIRT group had a partial glossectomy with ipsilateral neck dissection. | Survival events were per standard definitions.   The diagnosis of mandibular ORN or STN was made if there was non-healing exposed bone or soft tissue necrosis for 3-6 months without evidence of clinically recurrent tumor.  The Kaplan-Meier method was used in the survival analyses. | Pertinent pt and disease variables were summarized and accounted for in the analyses and discussion. | The FU schedule and modalities were described adequately. the median FU length (74 months) was adequate for the reported outcome measures. All pts were accounted for. | The 6y survival and disease control rates for POIRT are excellent for a cohort of early OTSCC pts, and are statistically superior to the exclusive HDR BRT group.  For the POIRT cohort, the 6y local RFS and nodal RFS were 100% and 96.2%. The 6y OS and DFS were 92.3% and 92.3%, respectively.  For the exclusive HDR BRT cohort, the 6y local RFS and nodal RFS were 85.1% and 65.5%. The 6y OS and DFS were 74.7% and 55.3%, respectively.  The local recurrence rates following surgery alone for tongue primary range from 10-63% as reported in contemporary series.  No pt developed STN or ORN in the POIRT group, compared to 16% and 7% in the exclusive HDR BRT group. | CIs were not reported.   The sample size (n=26) allows for estimates with low precision. | The 6y survival and disease control rates are a credible improvement on outcomes with exclusive HDR BRT or surgery alone. | The results are compatible with the notion that adjuvant radiotherapy limited to the tumor bed volume after wide resection for early OTSCC may improve outcomes with radiotherapy or surgery alone. | The surgical and BRT techniques are current. | The study provides retrospective data on long-term survival and toxicity rates with POIRT for resected early OTSCC with close or positive margins. |
| Teudt 2014 | **Low risk** | **Low risk** | **High risk** | **Low risk** | **High risk** | **Low risk** | **Uncertain risk** | **High risk** | **Low risk** | **Low risk** | **High risk** | **Low risk** |
|  | The study retrospectively evaluated the locoregional control, survival and toxicity outcomes of multi-modality treatment combining less aggressive surgery and POIRT ± EBRT and ChT for , nonmetastatic SNCs. | Between January 2006 and January 2013, 35 pts with SNCs were treated with less aggressive surgery and POIRT ± EBRT and ChT. Twenty-two had primary disease, for which separate survival outcomes were reported . | The surgery and BRT protocols were described adequately.  The extent of surgery was highly individualized, with aims of maximal resection with function preservation, and reconstruction as necessary.  The EBRT, the use of ChT, and the sequence and combination of surgery + POIRT, EBRT, and ChT, were also highly individualized. | Survival events were per standard definitions.   Toxicity was evaluated per standard criteria (CTCAE).  The Kaplan-Meier method was used in the survival analyses. | Pertinent pt and disease variables were summarized and accounted for in the analyses and discussion.  Different histologies were allowed, for which prognoses in terms of disease control and survival differ.  Co-interventions - ChT, EBRT - were allowed, on an individualized basis. | The FU schedule and modalities were described adequately. the median FU length (28 months) was adequate for the reported outcome measures. All pts were accounted for. | The 3y LC, RC and DC rates for pts with primary disease were 91%, 95% and 95%, respectively. The estimated 3y DFS was 83%.  Different histologes were allowed, for which prognoses in terms of disease control and survival differ.  Overall complication rate was 57% for the entire cohort, including recurrent disease with or without prior irradiation. | CIs were not reported.   The sample size (n=22) allows for estimates with low precision. | The 3y LC, RC, DC and DFS are excellent and seem attainable for a cohort where GTR (R0, R1) was achieved in the the majority (~89%), and appropriate adjuvant treatment given per histology, resection status, and stage.  The reported complication rate is high but is compatible given the complexity of the surgery and the co-interventions. | The results are compatible with a 5y OS estimate of 49.7% to 56.4% in a contemporary cohort of 6739 pts with SNCs treated with surgery, EBRT, or both (Turner, 2012). | The extent of surgery was highly individualized, with aims of maximal resection with function preservation, and reconstruction as necessary.  The EBRT, the use of ChT, and the sequence and combination of surgery + POIRT, EBRT, and ChT, were also highly individualized.  All the above may require a highly specialized team to replicate. | The study provides retrospective data on medium-term disease control, survival and toxicity rates with POIRT ± EBRT ± ChT for resected, , nonmetastatic primary SNCs. |
| **ChT**, chemotherapy; **CI**, confidence interval; **CTCAE**, Common Terminology Criteria for Adverse Events; **DC**, distant control; **DFS**, disease-free survival; **DSS**, disease-specific survival; **EBRT**, external beam radiotherapy; **EORTC**, European Organisation for Research and Treatment of Cancer; **FU**, follow up; **GTR**, gross total resection; **HDR**, high dose rate; **HNC**, head and neck cancer; **HNSCC**, head and neck squamous cell carcinoma; **IRT** interventional radiotherapy; **LC**, local control; **LRC**, locoregional control; **ORN**, osteoradionecrosis; **OS**, overall survival; **OTSCC**, oral tongue squamous cell carcinoma; **POIRT**, peri-operative interventional radiotherapy; **pt**, patient; **R0**, negative margins; **R1**, positive margins; **RC**, regional control; **RFS**, recurrence-free survival; **RTOG**, Radiation Therapy Oncology Group; **SCC**, squamous cell carcinoma; **SNC**, sinonasal cancer; **STN**, soft tissue necrosis; **UICC**, Union for International Cancer Control; **v**, version; **y**, year | | | | | | | | | | | | |

| **Peri-operative interventional radiotherapy in the re-irradiation setting** |
| --- |

| **Study ID** | **Research Question** | **Selection bias** | **Measurement bias (exposure)** | **Measurement bias (outcomes)** | **Confounding factors** | **Follow-up** | **Magnitude of effect** | **Precision of estimate** | **Credibility** | **Empiric congruence** | **Applicability** | **Implications to Practice** |
| --- | --- | --- | --- | --- | --- | --- | --- | --- | --- | --- | --- | --- |
| **Non-controlled clinical trial** | | | | | | | | | | | | |
| Martínez-Fernández, 2017 | **Low risk** | **Low risk** | **High risk** | **Low risk** | **High risk** | **Low risk** | **Low risk** | **High risk** | **Low risk** | **Low risk** | **High risk** | **Low risk** |
|  | The study prospectively assessed the disease control, survival, and complication rates with POIRT for resected, nonmetastatic primary or recurrent HNSCC with prior irradiation. | From February 2001 to November 2015, 63 patients with primary or recurrent HNC with prior irradiation were recruited into the study. Of these, 24% had second primaries, 76% had recurrences.  The majority (95%) were SCC. | The surgery and POIRT protocols were described adequately.  All patients had a GTR. EBRT and ChT were not given in the salvage setting.  More than 10% (20.6%) was treated off-protocol due to toxicity. | Survival events were per standard definitions.   Toxicity criteria was evaluated per standard criteria (RTOG acute/late radiation morbidity scoring schema).  Toxicities strongly related to the surgery and that occurred before completion of the POIRT were excluded from the POIRT toxicity analysis.  The Kaplan-Meier method was used in the survival analyses. | Time to recurrence and time to reirradiation, which are important factors to disease control and toxicity outcomes, were not discussed. | The FU schedule and modalities were described adequately. the median FU length (81.6 months) was adequate for the reported outcome measures. All patients were accounted for. | The 5y and 10y disease control, survival and toxicity rates are acceptable, given a cohort of patients with mostly recurrent disease and prior irradiation.   The 5y LRC and OS were 55% and 35.6%, respectively; the 10y LRC and OS were 55% and 29.1%.  The 10y cumulative incidence of grade 3-4 acute and late toxicity were 17.5% and 42.9%. The 10y cumulative incidence of grade 5 acute and late toxicity were 6.3% and 1.6%. | CIs were not reported.   The sample size (n=63) allows for estimates with moderate precision. | The 5y and 10y disease control, survival and toxicity rates are consistent with those reported in the only other contemporary series with long-term outcomes, that is included in this review (Pellizzon, 2006). | The results are compatible with the notion that adjuvant reirradiation limited to the tumor bed volume after wide resection for recurrent HNC after prior irradiation may be associated with long-term survival at the cost of significant late toxicity. | The surgical technique was highly individualized, to achieve GTR, and to reconstruct as necessary. This may require a highly specialized team to replicate. | The study provides prospective data on the long-term disease control, survival, and complication rates with POIRT for resected, nonmetastatic primary or recurrent HNC with prior irradiation. |
| **Retrospective cohort** | | | | | | | | | | | | |
| Bussu 2024 | **Low risk** | **Low risk** | **Low risk** | **Low risk** | **High risk** | **Low risk** | **High risk** | **High risk** | **Low risk** | **Low risk** | **High risk** | **Low risk** |
|  | The study retrospectively evaluated the medium-term locoregional control, survival and toxicity outcomes with POIRT and with exclusive HDR BRT in patients with nonmetastatic, recurrent HNC with prior irradiation. | Between December 2010 and June 2023, patients with recurrent HNC were treated with POIRT or exclusive HDR BRT if unresectable. The POIRT group included 29 patients. | The surgery and BRT protocols were described adequately.  All patients in the POIRT group had a GTR. | Survival events were per standard definitions.   Toxicity was evaluated per standard criteria (CTCAE) v5.0.  The Kaplan-Meier method was used in the survival analyses. | Time to recurrence and time to reirradiation, which are important factors to disease control and toxicity outcomes, were not discussed.  Two patients had second or third re-irradiation. | The FU schedule and modalities were described adequately. the median FU length (24.5 months) was adequate for the reported outcome measures. All patients were accounted for. | The 2y survival and disease control rates are acceptable, and toxicity rates are excellent for a cohort of recurrent HNC patients with prior irradiation.  The 2y local RFS, DSS and OS were 29.4%, 49.85%, and 46.3%, respectively. The acute toxicity rate is 3.4% (grade 1-2 ) with no grade ≥3 events.   Time to recurrence and time to reirradiation could give important context to the above rates. | CIs were not reported.   The sample size (n=29) allows for estimates with low precision. | The 2y disease control, survival and toxicity rates are consistent with outcomes reported in the few contemporary series that are available and are included in this review (Teudt, 2014; Rudzianskas, 2012). | The results are compatible with the notion that adjuvant reirradiation limited to the tumor bed volume after wide resection for recurrent HNC after prior irradiation may improve tolerance and toxicity of salvage treatment. | The surgical technique was highly individualized, to achieve GTR, and to reconstruct as necessary. This may require a highly specialized team to replicate. | The study provides retrospective data on medium-term disease control, survival and toxicity rates with POIRT for resected, nonmetastatic recurrent HNSCC with prior irradiation. |
| Soror 2023 | **Low risk** | **High risk** | **High risk** | **Low risk** | **High risk** | **High risk** | **High risk** | **High risk** | **High risk** | **Low risk** | **High risk** | **Low risk** |
|  | The study retrospectively evaluated the medium-term locoregional control, survival and toxicity outcomes with POIRT with or without EBRT, in patients with nonmetastatic, recurrent HNC with or without prior irradiation. | Between January 2016 and June 2020, patients with recurrent HNC with or without prior irradiation were treated with POIRT with or without EBRT.   The POIRT group included 60 patients, of which 70% had prior irradiation. Separate outcomes were not reported for those with prior irradiation. | The surgery and BRT protocols were described adequately.  The extent of surgery was highly individualized, with aims of radical resection or organ preservation, and pedicled or free flap reconstruction as necessary. | Survival events were per standard definitions.   Toxicity was evaluated per standard criteria (CTCAE) v5.0.  The Kaplan-Meier method was used in the survival analyses. | Time to recurrence and time to reirradiation, which are important factors to disease control and toxicity outcomes, were not discussed. | The FU schedule and modalities were described adequately. However, the median FU length (22.4 months) is short for the reported outcome measures. All patients were accounted for. | The 3y recurrence-free survival is excellent, and toxicity rates are acceptable for a cohort of recurrent HNC patients, the majority with prior irradiation.  The 3y and 5y RFS were 88.1% and 37.3%, respectively. The 3y and 5y OS were 39.2%, and 16.6%, respectively.   The authors have not explained the discrepancy between the 3y RFS and 3y OS. No grade 5 toxicities were reported. | CIs were not reported.   The sample size (n=60) allows for estimates with moderate precision. The toxicity estimates may be higher for the re-irradiation subset (70%). | The 3y and 5y OS rates are lower than the 3y and 5y local RFS. By definition, local RFS rates should be lower than or equal to OS rates. | The results are compatible with the notion that adjuvant reirradiation limited to the tumor bed volume combined with individualized wide-field irradiation doses after wide resection for recurrent HNC after prior irradiation may be associated with excellent disease control and acceptable medium-term toxicity. | The surgical technique was highly individualized, to achieve GTR or preserve organ, and to reconstruct as necessary. This may require a highly specialized team to replicate. | The study provides retrospective data on medium-term disease control, survival and toxicity rates with POIRT for resected, nonmetastatic recurrent HNSCC with prior irradiation. |
| Ritter 2016 | **Low risk** | **High risk** | **High risk** | **Low risk** | **Low risk** | **High risk** | **Low risk** | **Low risk** | **Uncertain risk** | **Uncertain risk** | **High risk** | **Low risk** |
|  | The study retrospectively compared the locoregional control, survival and toxicity outcomes of multi-modality treatment combining surgery and POIRT ± EBRT in 18 patients with nonmetastatic, recurrent HNCs treated with cetuximab and taxane and 18 matched-pair controls.  Secondarily, the study reported toxicity outcomes with POIRT ± EBRT in a bigger population of patients with nonmetastatic, recurrent HNCs, from which the cetuximab-taxane group and the matched-pair controls were selected. | Between January 2006 and May 2013, 94 patients with recurrent HNCs were treated with surgery and POIRT ± EBRT and ChT.   93.6% had surgery and POIRT; 4.4% were unresectable and had HDR BRT as part of definitive irradiation.   67% had prior irradiation. Separate outcomes were not reported for those with prior irradiation.  47.9% had several recurrences and multiple courses of prior irradiation. | The BRT protocols were described adequately.  The intent of surgery (GTR, maximal resection with organ preservation, or palliative debulking) was not stated. 8.5% did not undergo surgery.  The extent of surgery was likely individualized, given the multiple HNC sites included. | Survival events were per standard definitions.   Toxicity was evaluated per standard criteria (Common Terminology Criteria of the German Cancer Society; LENT SOMA).  The Kaplan-Meier method was used in the survival analyses. | Pertinent patient and disease variables, including time to recurrence, were summarized and accounted for in the analyses and discussion. | The FU schedule and modalities were described adequately. the mean FU length (13.4 months) is short. All patients were accounted for. | The survival outcomes were not reported for the population of interest and was reported only for the study group (cetuxilab-taxane) and the matched-pair control.  Toxicity rates are acceptable given a cohort of which 67% had prior irradiation. | CIs were not reported.   The sample size (n=94) allows for estimates with moderate to high precision. Toxicity rates may be higher for a purely re-irradiation cohort. | Overall toxicity incidence rates fall within the range reported by contemporary series. However, separate rates for acute and chronic toxicity were not reported. | The survival outcomes were not reported for the population of interest. separate rates for acute and chronic toxicity were not reported. | The surgical technique was likely highly individualized and may require a highly specialized team to replicate. | The study provides retrospective data on short-term toxicity rates with POIRT ± EBRT for resected, nonmetastatic recurrent HNCs with or without prior irradiation. |
| Teudt 2014 | **Low risk** | **Uncertain risk** | **High risk** | **Low risk** | **High risk** | **Low risk** | **Uncertain risk** | **High risk** | **Uncertain risk** | **Uncertain risk** | **High risk** | **Low risk** |
|  | The study retrospectively evaluated the locoregional control, survival and toxicity outcomes of multi-modality treatment combining less aggressive surgery and POIRT ± EBRT and ChT for nonmetastatic SNCs. | Between January 2006 and January 2013, 35 patients with SNCs were treated with less aggressive surgery and POIRT ± EBRT and ChT. Thirteen had recurrent disease; it was not specified how many of these had prior irradiation. | The surgery and BRT protocols were described adequately.  The extent of surgery was highly individualized, with aims of maximal resection with function preservation, and reconstruction as necessary.  The EBRT, the use of ChT, and the sequence and combination of surgery + POIRT, EBRT, and ChT, were also highly individualized. | Survival events were per standard definitions.   Toxicity was evaluated per standard criteria (CTCAE).  The Kaplan-Meier method was used in the survival analyses. | Time to recurrence and time to reirradiation, which are important factors to disease control and toxicity outcomes, were not discussed.  Different histologes were allowed, for which prognoses in terms of disease control and survival differ.  Co-interventions - ChT, EBRT - were allowed, on an individualized basis. | The FU schedule and modalities were described adequately. the median FU length (28 months) was adequate for the reported outcome measures. All patients were accounted for. | The 3y LC and DFS rates of 30% and 34%, respectively, are acceptable for a cohort of recurrent HNCs.  Separate toxicity rates were not reported for the recurrent group. | CIs were not reported.   The sample size (n=13) allows for estimates with low precision. | The 3y LC and DFS rates are consistent with other series included in this review (Bussu, 2024).   Separate toxicity rates were not reported for the recurrent group. | The results are compatible with the notion that adjuvant reirradiation limited to the tumor bed volume after wide resection for recurrent HNC after prior irradiation may afford acceptable disease control and survival rates.  Separate toxicity rates were not reported for the recurrent group. | The extent of surgery was highly individualized, with aims of maximal resection with function preservation, and reconstruction as necessary.  The EBRT, the use of ChT, and the sequence and combination of surgery + POIRT, EBRT, and ChT, were also highly individualized.  All the above may require a highly specialized team to replicate. | The study provides retrospective data on medium-term disease control, survival and toxicity rates with POIRT ± EBRT ± ChT for resected, nonmetastatic recurrent SNCs with or without prior irradiation. |
| Rudzianskas 2012 | **Low risk** | **Low risk** | **Low risk** | **Low risk** | **Low risk** | **Low risk** | **Low risk** | **High risk** | **Low risk** | **Uncertain risk** | **Low risk** | **Low risk** |
|  | The study retrospectively evaluated the disease control, survival and toxicity outcomes with POIRT and with exclusive HDR BRT in patients with nonmetastatic recurrent HNSCC with prior irradiation. | From December 2008 to March 2010, 30 patients with recurrent HNSCCs with prior irradiation were treatec with POIRT or exclusive HDR BRT. Thirteen had POIRT. Separate outcomes were reported for the POIRT group. | The surgery and BRT protocols were described adequately. | Survival events were per standard definitions.   Toxicity criteria was evaluated per standard criteria (RTOG acute/late radiation morbidity scoring schema).  The Kaplan-Meier method was used in the survival analyses. | Pertinent patient and disease variables, including time to recurrence, were summarized and accounted for in the analyses and discussion. | The FU schedule and modalities were described adequately. the median FU length (28 months) was adequate for the reported outcome measures. All patients were accounted for. | The 2y LC and OS rates of 77% and 62%, are excellent for a cohort of recurrent HNSCCs.  Toxicity rates are low, but not reported separately for the POIRT group. | CIs were not reported.   The sample size (n=13) allows for estimates with low precision. | The 2y LC and OS rates are consistent with, if not better than, that reported by other series included in this review (Bussu, 2024; Teudt 2014).   Toxicity rates are low, but not reported separately for the POIRT group. | The results are compatible with other studies showing that POIRT for HNSCC may afford better disease control and survival rates, compared to HDR BRT alone (Potharaju, 2018).  Separate toxicity rates were not reported for the POIRT group. | The surgical, BRT and EBRT techniques are current.   Oral cavity and oropharyngeal tumors were not resected and treated with exclusive HDR BRT. | The study provides retrospective data on medium-term disease control and survival rates with POIRT for resected, nonmetastatic recurrent HNSCCs with prior irradiation. |
| Pellizzon 2006 | **Low risk** | **High risk** | **High risk** | **High risk** | **Low risk** | **High risk** | **Low risk** | **High risk** | **Low risk** | **Low risk** | **Low risk** | **Low risk** |
|  | The study retrospectively evaluated the disease control, survival and toxicity outcomes with POIRT + EBRT in patients with node-only HNSCC recurrences with or without prior irradiation. | Between October 1994 and June 2004, 21 patients with node-only HNSCC recurrences were treated with POIRT + EBRT.   Only fifteen (71.4%) had prior irradiation. Outcomes were not reported separately for the reirradiation group. | The surgery and BRT protocols were described adequately.   The dose of the second course of EBRT was individualized according to the dose of the first irradiation. | Survival events were per standard definitions.   Toxicity was not evaluated per standard criteria.  The Kaplan-Meier method was used in the survival analyses.   Outcomes were not reported separately for those with prior irradiation. | Pertinent patient and disease variables, including time to alvage therapy, were summarized and accounted for in the analyses and discussion. | The median FU (36 months) is short for 5y and 8y survival estimates.   All patients were accounted for. | The 5y RFS and OS rates of 42.5% and 50% are acceptable for patients with recurrent HNSCC.  A cumulative incidence of 19.4% for grade 3-4 late toxicity is acceptable for a cohort that is mostly previously irradiated. | CIs were not reported.   The sample size (n=21) allows for estimates with low precision. | The 5y disease control, survival and toxicity rates are consistent with those reported in the only other contemporary series with long-term outcomes, that is included in this review (Martínez-Fernández, 2017). | The results are compatible with the notion that adjuvant reirradiation limited to the tumor bed volume combined with individualized wide-field irradiation doses after wide resection for recurrent HNC after prior irradiation may be associated with long-term survival at the cost of significant late toxicity. | The surgical, BRT and EBRT techniques are current.   The surgery entailed neck dissections. | The study provides retrospective data on long-term disease control, survival and toxicity rates with POIRT + EBRT for resected, node-only HNSCC recurrences with or without prior irradiation. |
| **IRT** interventional radiotherapy; **ChT**, chemotherapy; **CI**, confidence interval; **CTCAE**, Common Terminology Criteria for Adverse Events; **DC**, distant control; **DFS**, disease-free survival; **DSS**, disease-specific survival; **EBRT**, external beam radiotherapy; **EORTC**, European Organisation for Research and Treatment of Cancer; **FU**, follow up; **GTR**, gross total resection; **HDR**, high dose rate; **HNC**, head and neck cancer; **HNSCC**, head and neck squamous cell carcinoma; **LC**, local control; **LRC**, locoregional control; **ORN**, osteoradionecrosis; **OS**, overall survival; **OTSCC**, oral tongue squamous cell carcinoma; **POIRT**, peri-operative interventional radiotherapy; **pt**, patient; **R0**, negative margins; **R1**, positive margins; **RC**, regional control; **RFS**, recurrence-free survival; **RTOG**, Radiation Therapy Oncology Group; **SCC**, squamous cell carcinoma; **SNC**, sinonasal cancer; **STN**, soft tissue necrosis; **UICC**, Union for International Cancer Control; **v**, version; **y**, year | | | | | | | | | | | | |
