# Supplementary material for: Local Control, Survival, and Toxicity Outcomes with High-Dose-Rate Peri-Operative Interventional Radiotherapy (Brachytherapy) in Head and Neck Cancers: A Systematic Review
Source: J Pers Med. 2024 Aug 11;14(8):853. doi: 10.3390/jpm14080853 (PMC11355512; doi:10.3390/jpm14080853)
Supplement: Supplementary file 1 [file jpm-14-00853-s001.zip › S3 Detailed Study Population and Intervention Characteristics.docx]

**Table 2. Study Information and Population, and Intervention Groups and Characteristics**

| **Peri-operative interventional radiotherapy in the primary setting** | | | | | | | | | | | | | | | | |
| --- | --- | --- | --- | --- | --- | --- | --- | --- | --- | --- | --- | --- | --- | --- | --- | --- |
| **Study Information** | | | | **Patient Characteristics** | | | | | | **Intervention Characteristics** | | | | | | |
| **Study ID (References)** | **Country** | **Study Period** | **Eligibility** | **n**  **/Subsets, %** | **M/F** | **Mdn Age**  **(Range)** | **Site, %** | **Histology and Grade, %** | **Stage, %** | **Resection/ Margin Status, %**  **/Reconstruction, %** | **EBRT, %**  **/Dosing**  **/Technique** | **BRT Dosing** | **Implant Technique and CTV** | **Dosimetry CTV and Constraints** | **Start of BRT  (day post-op)** | **Chemo, % /Regimen, %** |
| **Non-controlled clinical trials** | | | | | | | | | | | | | | | | |
| **Ianovski, 2020**  (Ianovski, 2020) | Canada | Sep 2009 to  Apr 2017 | *Inclusion*  T1-3 N0-3 M0 OTSCC Primary tumor amenable to WLE Adequate mandible clearance MDT-validated  *Exclusion*  Zubrod PS>2 Prior HN RT  Prior chemo Previous cancer except NMSC  Other treatment for HNC Active infection | 55  *POBT* indicated and given, 75  *(POBT not indicated*, 25) | *Entire cohort*  0.90 | *Entire cohort*  62  (24-92) | *Entire cohort*  OT, 100 | *Entire cohort* SCC, 100  G1, 49 G2, 38 G3, 13 | *Entire cohort*  pT1, 49 pT2, 47 pT3, 4 pT4, 0  pN0, 65 pN1, 15 pN2, 20 | *POBT subset* R0, 0 Close (2.1-5mm), 58 R1, 42  Recon*,* 100 | *POBT subset*, 39  Involved neck, 55Gy/25F Uninvolved neck, 50Gy/25F  BRT 50% isodose limited to Dmax 25Gy  IMRT, VMAT | Close margins 34Gy/10F, 63  R1  40.8Gy/12F, 37 | ISBT  CTV: Tumor bed | CTV: 5mm around the catheters | D3-5 | *POBT subset*  34  If ENE, RT + concurrent weekly carboplatin 100mg/m2 + taxol 40mg/m2 |
| **Gaztañaga, 2012**  (Gaztañaga, 2012  Martínez-Monge, 2011  Martínez-Monge, 2009) | Spain | Oct 2000 to  Oct 2008 | *Inclusion*  HNSCC primary or recurrent GTR M0 per Chest CT ±PET | 57  *Primary*, 70  *(Recurrent*,  30) | *Primary subset*  2.17 | *Entire cohort*  59  (25-85) | *Primary subset* OC, 52 (OT, 35; FOM, 10; Gingiva, 4; Other, 5)  OPx, 21  HPx, 7  Neck, 18  (Source of metastasis: NC, 2  Lx, 9  Skin or scalp, 5  Unknown 2) | *Primary subset*  SCC, 100  G1, 17 G2, 60 G3, 23 | *Primary subset* cN0, 21 cN1-2, 79  pN0, 30 pN+, 63 pNx, 7 | *Entire cohort*  R0 (10mm), 12 Close (Mdn 3.0 mm), 35 R1, 53 | *Entire cohort*  100  45Gy/25F | R0  32Gy/8F BID 6h apart  R1  40Gy/10F BID 6h apart | ISBT CTV: Tumor bed and all surgical bed considered recurrence risk category 2 (≥2 nodes or ENE) or 3 (R1) | CTV: Tumor bed and high-risk volumes  2D (2001-2006): ICRU58 MTD 3D (2006 onwards): CTV D90  DHI≥0.6 DHI = TV100-TV150/TV100 | D2-3 | *Primary subset* 63  Cisplatin-paclitaxel, 60 Cisplatin-other, 4 |
| **Retrospective cohort** | | | | | | | | | | | | | | | | |
| **Potharaju, 2018**  (Potharaju, 2018) | India | Jan 2000 to  Sep 2010 | *Inclusion*  OTSCC cT1-T2N0M0 (AJCC 2009)  Had WLE + ipsilateral MRND No neck EBRT Minimum FU 6 years  M0 per HN CT/US  *Exclusion*  Projected CTV bed >1cm, requiring >1 plane implant | 73  *POBT*, 36  *(Definitive IRT,* 64) | *Entire cohort*  2.25 | *Entire cohort*  52 | *Entire cohort*  OT, 100 | *Entire cohort*  SCC, 100 | *Entire cohort*  T1, 14 T2, 12  N0, 100 | <5mm, x ≥5-10mm, x  Recon, 0 | None | 40Gy/10F BID 6h apart | ISBT, single-plane  CTV: Tumor bed | CTV: 5mm around catheters  2D, 77 3D, 23 | D5-7 | None |
| **Teudt, 2014**  (Teudt, 2014) | Germany | Jan 2006 to  Jan 2013 | Primary or recurrent SNC  Had surgery + POBT ± EBRT/CRT MDT-validated for MSR + function preservation + optimal POBT implant M0 per chest x-ray/CT, upper abdominal US/CT | 35  *Primary*, 63  *(Recurrent,*  *37)* | *Entire cohort*  2.89 | *Entire cohort*  60 | *Entire cohort*  NC, 46 PNS, 54 | *Entire cohort*  SCC, 63 Adeno, 20 Melanoma, 6 ACC, 3 TCC, 3 Hemangiopericytoma, 3  Unspecified, 3  G1, 9  G2, 37 G3, 49  GX, 6 | *Entire cohort* UICC I, 17  UICC II, 20  UICC III, 11 UICC IV, 51 | *Entire cohort* R0, 54  R1, 31 R2, 3  Rx, 11  Osteosynthesis plates as needed | *Entire cohort*  57  Mdn 50.4Gy (40-63Gy) | *Entire cohort*  Mdn 20Gy (10-35Gy)/ 2.5Gy-F BID 6h apart | ISBT  Intensity-modulation by variable catheter spacing (5-12mm) | CTV: Maximum 10mm around catheters | Mdn D7 (D2-14) | *Entire cohort,* 31 (chemo given only for SCC)  Cisplatin, 26 Taxane, 9 Etoposide, 3 |
| **2D,** two dimensional; **3D,** three dimensional; **Adeno,** adenocarcinoma; **ACC,** adenoid cystic carcinoma; **BID,** twice daily; **c,** clinical; **CRT,** chemoradiation; **CT,** computed tomography; **CTV,** clinical target volume; **D,** day; **D90**, dose received by 90% of the volume; **DHI**, dose homogeneity index; **EBRT**, external beam radiotherapy; **ENE**, extranodal extension; **F**, fraction; **FOM**, floor of mouth; **FU**, follow up; **G**, grade; **GTR**, gross total resection; **Gy**, Gray; **h**, hour; **HN**, head and neck; **HNC**, head and neck cancer; **HNSCC**, head and neck squamous cell carcinoma; **HPx**, hypopharynx; **ICRU**, International Commission on Radiation Units and Measurements; **IMRT**, intensity-modulated radiotherapy; **IRT,** interventional radiotherapy; **ISBT**, interstitial interventional radiotherapy; **Lx**, larynx; **M**, metastasis stage; **Mdn**, median; **MDT**, multidisciplinary team; **MSR**, maximal safe resection; **MTD**, minimum target dose; **N**, nodal stage; **NC**, nasal cavity; **NMSC**, non-melanoma skin cancer; **OC**, oral cavity; **OPx**, oropharynx; **OT**, oral tongue; **OTSCC**, oral tongue squamous cell carcinoma; **PET**, positron emission tomography; **PNS**, paranasal sinus; **POBT**, perioperative interventional radiotherapy; **p**, pathologic; **PS**, performance status; **R**, resection; **RT**, radiotherapy; **SCC**, squamous cell carcinoma; **SNC**, sinonasal cancer; **T**, primary tumor stage; **TCC**, transitional cell carcinoma; **TV**, target volume; **UICC**, Union for International Cancer Control; **US**, ultrasound; **VMAT**, volumetric arc therapy; **WLE**, wide local excision; **x**, unknown | | | | | | | | | | | | | | | | |

| **Peri-operative interventional radiotherapy in the re-irradiation setting** | | | | | | | | | | | | | | | | | |
| --- | --- | --- | --- | --- | --- | --- | --- | --- | --- | --- | --- | --- | --- | --- | --- | --- | --- |
| **Study Information** | | | | **Patient Characteristics** | | | | | | | **Intervention Characteristics** | | | | | | |
| **Study ID (References)** | **Country** | **Study Period** | **Eligibility** | **n**  **/Subsets, %** | **M/F** | **Mdn Age**  **(Range)** | **Site, %** | **Histology and Grade, %** | **Setting and Stage, %** | **Prior RT, %**  **/Setting, Dosing /Chemo /Time to ReRT** | **Resection/ Margin Status, %**  **/Reconstruction, %** | **EBRT, %**  **/Dosing**  **/Technique** | **BRT Dosing** | **Implant Technique and CTV** | **Dosimetry CTV and**  **Constraints** | **Start of BRT  (day post-op)** | **Chemo, %**  **/Regimen, %** |
| **Non-controlled clinical trial** | | | | | | | | | | | | | | | | | |
| **Martínez-Fernández, 2017**  (Martínez-Fernández, 2017, Gaztañaga, 2012  Martínez-Monge, 2011  Martínez-Monge, 2006) | Spain | Feb 2001to Nov 2015 | HNC recurrence or second primary  Biopsy-proven Prior irradiation  Planned curative surgery  M0 per Chest CT or PET | 63 | 2.7 | 63 (26-82) | Neck, 32  OT, 24  BOT, 13  OPx, 8  Lower gum/FOM 6 HPx, 6  Parastomal, 5 SP/RMT, 3 Maxillary antrum, 2  Meningeal surface, 2 | SCC, 95  Adeno, 2 Undifferentiated carcinoma, 2  Lymphoepithelioma, 2  G1–2, 81 G3, 19 | *Second primary* 24% T1-2N0, 18 T3/N+, 6  *Recurrence*  76  (Mean 2.7cm, 0.6-8.0)  *Nodal status*  pN0, 38  pN+, 38 pNx, 24  ECE, 67 | 100  *EBRT*, 98 *IRT,* 14  *Prior surgery*, 64  *Chemo*  32 | R0 (10mm), 11 Close (Mdn 3.0 mm), 35 R1, 54 | None | ≤32Gy, 29 40Gy, 71  *R0*: 32Gy/8F BID 6h apart *R1:* 40Gy/10F BID 6h apart | ISBT  CTV: Tumor bed and all surgical bed considered recurrence risk category 2 (≥2 nodes or ENE) or 3 (positive margins) | CTV: Tumor bed and high-risk volumes  2D (2001-2006): ICRU58 MTD 3D (2006 onwards): CTV D90  4Gy prescribed to the CTVD90 if TV150 (6Gy isodose) <13 cc  Mandibular and vascular D10cc <4Gy | Mdn D4 (D0-D10) | None |
| **Retrospective cohort** | | | | | | | | | | | | | | | | | |
| **Bussu, 2024** (Bussu, 2024  Bussu, 2019  Tagliaferri, 2017) | Italy | Dec 2010 to Jun 2023 | *Inclusion*  Recurrent HNC Biopsy-proven  Prior irradiation ≥65Gy POBT or definitive BRT  MDT-validated M0 per PET-CT  *Exclusion*  Persistent disease post-RT Unresectable due to poor PS, carotid involvement, 3rd-4th recurrence, or heavy radiation fibrosis | 34  *POBT*, 85  *(Definitive IRT,* 15) | *POBT subset*  2.6 | *POBT subset* Mean 64.5 | *POBT subset*  *ICBT* NPx, 64  Ethmoid, 21 NC, 14  *ISBT* OC, 27  Lx, 20 HPx, 13  OPx, 13 Parotid, 7 Skin, 7 SMG, 7 Unknown primary, 7 | *POBT subset*  *ICBT* SCC, 72 Adeno, 14 ACC, 7 Melanoma, 7  *ISBT* SCC, 87  MMT, 7 Mucoepidermoid, 7 | *POBT subset*  *ICBT* LR, 100  (Second reRT, 3 Third reRT, 3)  *ISBT* LR, x% RR, x% | *POBT subset*  100  *ICBT*  Definitive, 64 Adjuvant, 36  *ISBT*  Definitive, 33 Adjuvant, 67  >65Gy, 100% | *POBT subset*  GTR, 100  *Recon*  *ICBT*, 7 *ISBT*, 87 | None | 30Gy/12F BID 6h apart | ISBT, ICBT CTV: Tumor bed and high-risk volumes | CTV: Tumor bed and high-risk volumes  CTV dose coverage optimized to respect OAR constraints per QUANTEC | D3-5 | *POBT subset*  *ICBT*, 21 *ISBT*, 0 |
| **Soror, 2023** (Soror, 2023) | Germany | Jan 2016to Dec 2020 | Recurrent HNC Biopsy-proven ± Prior irradiation Complete or partial resection  POBT ± EBRT MDT-validated M0 per neck US, radiographic or isotopic scans | 60  *ReRT*, 70  (*No prior RT*, 30) | *Entire cohort*  3.29 | *Entire cohort*  65.6  (15.4-92.7) | *Entire cohort*  OPx, 25  Neck, 23  OC, 23  Sinonasal/Orbit, 13  HPx, 5  NPx, 3  Ear, 3 | *Entire cohort*  SCC, 90  Adeno, 8 Other, 2 | *Entire* *cohort* LR, 68 RR, 23 Second primary, 8. | 70  Mdn 60Gy (32-70)  *Chemo*  45 | *Entire* *cohort*  R0, 32 Close margin (<5mm), 5 R1, 18 R2, 45  *Recon*  Pedicled or free flap, as indicated, x% | *Entire* *cohort*  12  30-50Gy | *Entire* *cohort*  Mdn 30Gy (12-40)/3Gy-F BID 6h apart | ISBT  CTV: Tumor bed + 15-20mm and high-risk volumes  8-12mm spacing | CTV: Tumor bed + 15-20mm and high-risk volumes | D2-5 | None |
| **Ritter, 2016** (Ritter, 2016) | Germany | January 2006  May 2013 | HNC recurrence  Biopsy-proven Planned curative surgery + POBT ± EBRT ± chemo  M0 | 94  *POBT*, 94 *ReRT*, 67  (*Definitive IRT,* 6  No prior RT, 33) | Not reported | *Entire cohort* <60, 38 ≥60, 62 | *Entire cohort* OPx/NPx, 28  OC, 26 Neck, 8  HPx/Lx, 6 Other 32 | *Entire cohort* SCC, 80 Other, 44  G1-2, 53 G3-4, 44 | *Entire cohort* UICC I-II, 33 UICC III-IV, 67  T1-2, 40 T3-4, 48 Tx, 10  N0, 71 N1-2, 22 N3, 3 | 67  Mdn 64.2 (33-105)( multiple recurrences/RT, 48)  *Chemo*  26  *Time to first recurrence*  Mdn 24mo (10-73)  <3mo, 10 ≤ 3mo, 84 | R0, 39 R1, 34 R2, 12 Rx, 6 No resection, 8  *Recon*  Pedicled, microvascular or random pattern flap, as indicated, x% | 26  Mdn 48.7Gy (30-60) | Mdn 25.9Gy (10-35)/2.5Gy (2.5-4.5) F BID 6h apart | ISBT | Intensity-modulation allowed for up to 200% within macroscopic tumor. OAR doses less than the reference isodose. |  | *Entire cohort*  16  Platinum, 5 Cetuximab-taxane, 19 |
| **Teudt, 2014** (Teudt, 2014) | Germany | January 2006  January 2013 | SNC primary or recurrence ± Prior irradiation MDT-validated for MSR + function preservation + optimal POBT implant M0 per chest x-ray/CT, upper abdominal US/CT | 35  *Recurrent*, 47  (*Primary*, 53) | *Entire cohort* 2.89 | *Entire cohort*  60 | *Entire cohort* NC, 46 PNS, 54 | *Entire cohort* SCC, 63  Adeno, 20 Melanoma, 6 ACC, 3  TCC, 3 Hemangiopericytoma, 3 Unspecified, 3  G1, 9  G2, 37 G3, 49  GX, 6 | *Entire cohort* UICC I, 17 UICC II, 20 UICC III, 11 UICC IV, 51 | Not reported | *Entire cohort* R0, 54 R1, 31 R2, 3  Rx, 11  *Recon*  Osteosynthesis plates as needed | *Entire cohort*  57  Mdn 50.4Gy (40-63Gy) | Mdn 20Gy (10-35Gy)/ 2.5Gy-F BID 6h apart | ISBT  CTV: Tumor bed  Intensity-modulation by variable catheter spacing (5-12mm) | CTV: Maximum 10mm around catheters | Mdn D7 (D2-14) | *Entire cohort*  31 (chemo given only for SCC)  Cisplatin, 26 Taxane, 9 Etoposide, 3 |
| **Rudzianskas, 2012** (Rudzianskas, 2012) | Lithuania | Dec 2008to Mar 2010 | HNSCC local or neck recurrence Biopsy-proven No bony invasion POBT or definitive BRT  Prior irradiation ± surgery ± chemo  M0  KPS ≥80 | 30  *POBT*, 43  (*Definitive IRT,* 57) | *Entire cohort* 2.33 | *Entire cohort*  59 (41-79) | *Entire cohort*  OC, 27 NC/PNS, 13 Parotid, 3  OPx, 13 Neck, 44 | *Entire cohort*  SCC, 100 | *Entire cohort*  LR, 57 RR, 43 | 100  *Definitive*, 33 EBRT, 13 CRT, 20  *Adjuvant*, 67 EBRT, 57 CRT, 10  Mdn 66 (50-72)  *Chemo* 30  *Time to first recurrence*  Mdn 12mo (3-19) | Not reported | None | 30Gy/12F BID 6h apart | ISBT  Catheter spacing 10-15mm | 3D: CTV D90  *Entire cohort* Mean CTV D90/fraction, 2.25Gy (1.9-2.5Gy)  V100, 69.53% (42-95) V150, 25.17% (14-44) V200, 13.32% (9-28)  Mean DHI, 0.66 (0.59-0.7) Mean DNR, 0.35 (0.28-0.37) |  |  |
| **Pellizzon, 2006** (Pellizzon, 2006) | Brazil | Oct 1994 to Jun 2004 | HNSCC with neck recurrence only  Biopsy-proven  Resectable ± Prior irradiation MDT-validated M0 per chest x-ray KPS≥60 | 21  *ReRT*, 71  (*No prior RT*, 29) | *Entire cohort* 3.2 | *Entire cohort* 53.5  (31-73) | *Entire cohort* Pharynx 48  OC, 29  Skin, 19  Neck, 5 | *Entire cohort* SCC, 100 | *Entire cohort*  RR 100 | 71  *Entire cohort*  Mdn 52Gy (30-66Gy)  *Chemo* 5  *Time to salvage therapy*  Mdn 32mo (14-86) | GTR, 100  *Recon*  As needed, x% | 100  *ReRT subset* Mdn 30Gy (25-50) | *ReRT subset* Mdn 24Gy | ISBT  CTV: Tumor bed + 15-20mm margins  Single plane, 90.5% Double plane, 9.5% | CTV: Tumor bed + 5mm  Catheter spacing 10-15mm  Dmax ≤135% Skin dose <60%  3D, x% 2D, x% | D5 (D4-D12) | *Entire cohort*  4.7%  Platinum |
| **2D,** two dimensional; **3D,** three dimensional; **Adeno,** adenocarcinoma; **ACC,** adenoid cystic carcinoma; **BID,** twice daily; **BOT,** base of tongue; **c,** clinical; **CRT,** chemoradiation; **CT,** computed tomography; **CTV,** clinical target volume; **D,** day; **D10cc**, dose to the most exposed 10cc volume; **D90**, dose received by 90% of the volume; **DHI**, dose homogeneity index; **Dmax**, maximum dose; **DNR**, dose non-uniformity ratio; **EBRT**, external beam radiotherapy; **ENE**, extranodal extension; **F**, fraction; **FOM**, floor of mouth; **FU**, follow up; **G**, grade; **GTR**, gross total resection; **Gy**, Gray; **h**, hour; **HN**, head and neck; **HNC**, head and neck cancer; **HNSCC**, head and neck squamous cell carcinoma; **HPx**, hypopharynx; **ICBT**, intracavitary interventional radiotherapy; **ICRU**, International Commission on Radiation Units and Measurements; **IMRT**, intensity-modulated radiotherapy; **IRT,** interventional radiotherapy; **ISBT**, interstitial interventional radiotherapy; **KPS,** Karnofsky Performance Status; **LR,** local recurrence; **Lx**, larynx; **M**, metastasis stage; **Mdn**, median; **MDT**, multidisciplinary team; **MMT,** malignant mixed tumor; **mo**, month; **MSR**, maximal safe resection; **MTD**, minimum target dose; **N**, nodal stage; **NC**, nasal cavity; **NMSC**, non-melanoma skin cancer; **NPx,** nasopharynx; **OAR**, organ at risk; **OC**, oral cavity; **OPx**, oropharynx; **OT**, oral tongue; **OTSCC**, oral tongue squamous cell carcinoma; **PET**, positron emission tomography; **PNS**, paranasal sinus; **POBT**, perioperative interventional radiotherapy; **p**, pathologic; **PS**, performance status; **QUANTEC**, Quantitative Analysis of Normal Tissue Effects in the Clinic; **R**, resection; **reRT**, reirradiation; **RMT**, retromolar trigone; **RR,** regional recurrence; **RT**, radiotherapy; **SCC**, squamous cell carcinoma; **SMG**, submandibular gland; **SNC**, sinonasal cancer; **SP**, soft palate; **T**, primary tumor stage; **TCC**, transitional cell carcinoma; **TV**, target volume; **UICC**, Union for International Cancer Control; **US**, ultrasound; **V100**, volume receiving 100% of the prescribed dose; **V150**, volume receiving 150% of the prescribed dose; **V200**, volume receiving 200% of the prescribed dose; **VMAT**, volumetric arc therapy; **WLE**, wide local excision; **x**, unknown | | | | | | | | | | | | | | | | | |
